# Evolutionary Specialization of a Promiscuous Designer Enzyme

**DOI:** 10.1021/acscatal.4c06409

**Published:** 2025-01-13

**Authors:** Reuben B. Leveson-Gower, Laura Tiessler-Sala, Henriette J. Rozeboom, Andy-Mark W. H. Thunnissen, Jean-Didier Maréchal, Gerard Roelfes

**Affiliations:** † Stratingh Institute for Chemistry, University of Groningen, 9747AG Groningen, The Netherlands; ‡ Insilichem, Departament de Química, Universitat Autònoma de Barcelona, 08193 Cerdanyola del Vallès, Spain; § Groningen Biomolecular Sciences and Biotechnology Institute, University of Groningen, 9747AG Groningen, The Netherlands

**Keywords:** Biocatalysis, Designer Enzymes, Noncanonical-Amino Acids, Directed Evolution, Quantum Chemistry, Molecular Dynamics, X-ray Crystallography

## Abstract

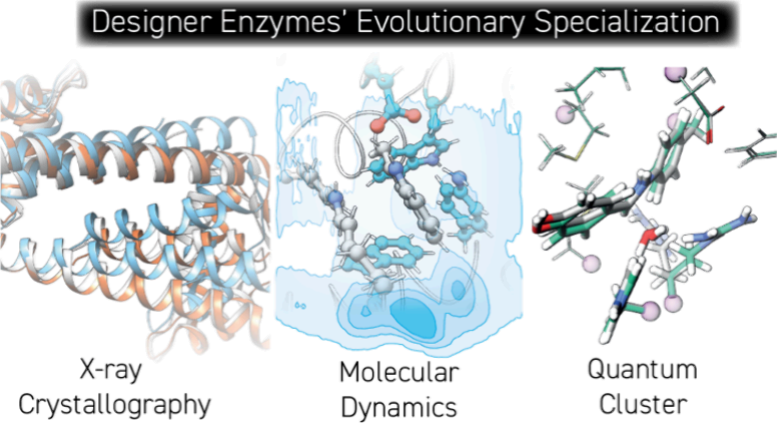

The evolution of a promiscuous enzyme for its various activities often results in catalytically specialized variants. This is an important natural mechanism to ensure the proper functioning of natural metabolic networks. It also acts as both a curse and blessing for enzyme engineers, where enzymes that have undergone directed evolution may exhibit exquisite selectivity at the expense of a diminished overall catalytic repertoire. We previously performed two independent directed evolution campaigns on a promiscuous designer enzyme that leverages the unique properties of a noncanonical amino acid (ncAA) *para*-aminophenylalanine (pAF) as catalytic residue, resulting in two evolved variants which are both catalytically specialized. Here, we combine mutagenesis, crystallography, and computation to reveal the molecular basis of the specialization phenomenon. In one evolved variant, an unexpected change in quaternary structure biases substrate dynamics to promote enantioselective catalysis, while the other demonstrates synergistic cooperation between natural side chains and the pAF residue to form semisynthetic catalytic machinery.

## Introduction

**Figure 1:**
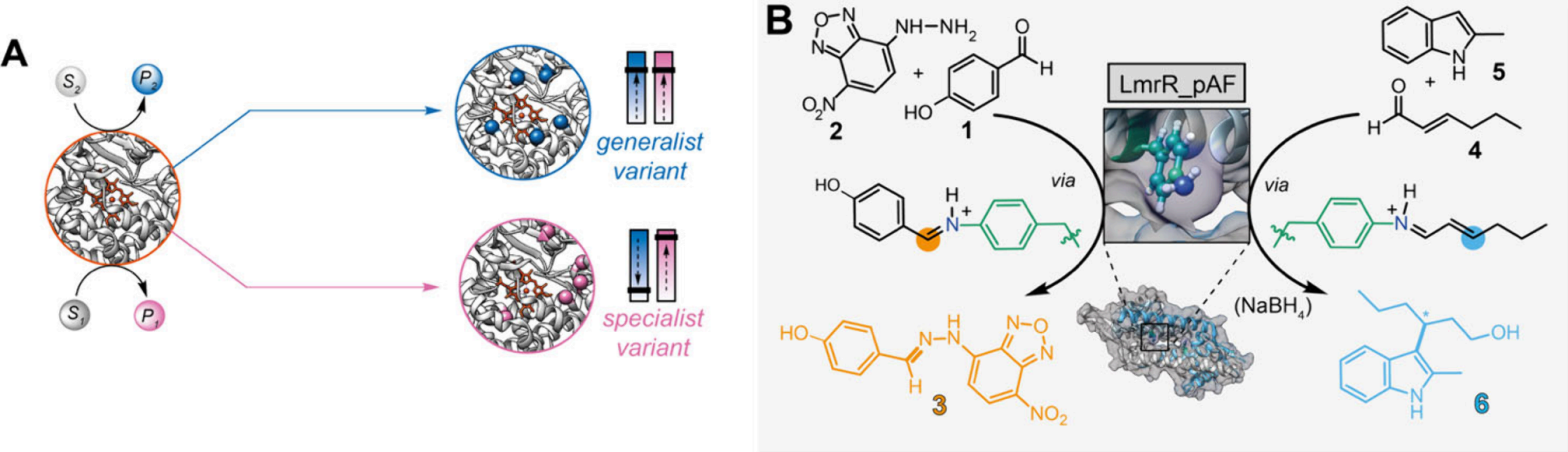
(A) Alternative outcomes of evolution of a promiscuous parental enzyme to produce either generalized (blue, top) or specialized (pink, bottom) evolved mutants. (B) Catalytic promiscuity of LmrR_pAF for hydrazone formation and Friedel–Crafts alkylation reactions.

Some enzymes possess the striking ability to catalyze multiple, mechanistically distinct, chemical transformations, a phenomenon known as catalytic promiscuity.^1,2^ This contrasts with the rigid specificity often exhibited by enzymes, e.g., to avoid toxic potential side-reactions.^3^ Catalytic promiscuity is thought to play a crucial role in the creation of new enzymatic activities in nature, which in turn can facilitate adaptation to new environmental pressures.^4,5^ Promiscuous enzymes are also privileged candidates for the discovery and engineering of new biocatalytic solutions for synthetic chemistry, being more likely to exhibit mechanistically related activities distinct from their native activity, and perhaps also being more prone to efficient evolutionary pathways.^6−8^ However, many chemical catalytic transformations exist where the mechanism bears scant similarity to any enzymatic chemistry, making the prospects of finding a suitable promiscuous enzyme for these transformations slim.^9,10^ Here, designer enzymes consisting of a protein scaffold equipped with an unnatural catalytic group, come into their own.^7,11^ The expanded palette of catalytic components confers the possibility for reaction mechanisms not seen in nature. Situations where designer enzymes exhibit catalytic promiscuity should be treated with particular importance, as they may allow the rapid expansion of the utility of these catalysts.^7,12^ During the evolution of such a promiscuous designer enzyme, its multiple activities could evolve concurrently or separately, resulting in generalist or specialist variants, respectively (Figure 1A). These phenomena are particularly important to enzyme engineers since the former may allow the development of multipurpose biocatalysts, while the latter could provide enzymes with strict selectivity needed for, e.g., late-stage functionalization in medicinal chemistry.^13^

Our group previously produced a promiscuous designer enzyme by incorporating the noncanonical amino acid *para*-aminophenylalanine at position valine-15 of the Lactococcal multidrug resistance regulatory (LmrR) protein scaffold.^14^ In doing so, we enabled the formation of iminium ion intermediates formed by the condensation of aldehydes with the amino moiety of the pAF catalytic residue, thus mimicking known reactivities of small molecule organocatalysts in a protein scaffold.^15^ So far, two distinct mechanistic pathways have been identified (Figure 1B): (I) transimination, enabling the reaction between benzaldehyde derivatives (e.g., **1**) and 4-hydrazino-7-nitro-2,1,3-benzoxadiazole (NBD-H, **2**)^14,16^ to afford the corresponding hydrazone (hydrazone formation reaction, e.g., **3**) and (II) conjugate addition, enabling the reaction between aliphatic α,β-unsaturated aldehydes (e.g., **4**) and indoles (e.g., **5**) to enantioselectively afford the corresponding Friedel–Crafts alkylated indole products (Friedel–Crafts alkylation (FC), e.g., **6**).^17,18^ For each of these activities, a directed evolution campaign was performed, resulting in an improved triple mutant for each reactivity. Assessment of the Michaelis–Menten kinetic parameters of these evolved mutants revealed that the mutations that improved one catalytic activity caused a concomitant decrease in the other, i.e., directed evolution produced catalytic specialization.^17^ In the present study, we combine mutagenic, crystallographic and computational studies to understand the molecular basis of this phenomenon, revealing a change in quaternary structure and direct cooperation between the ncAA and side-chains introduced during evolution underlying the specialization.

## Results and Discussion

**Figure 2:**
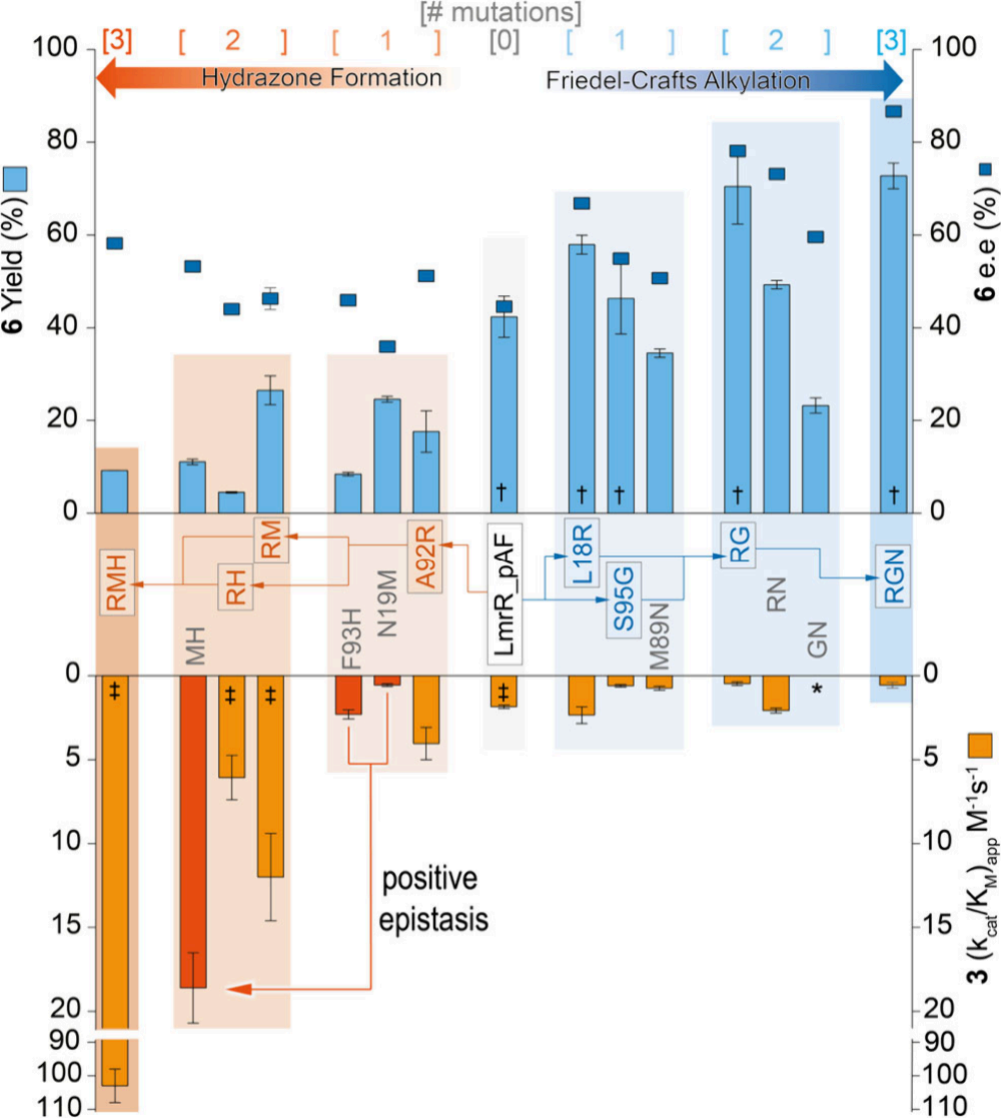
Evolutionary pathways of LmrR_pAF for its promiscuous hydrazone formation (orange) and Friedel–Crafts alkylation (blue) activities. The pathway followed during each directed evolution campaign by the respectively colored arrows, while mutants not explored in the pathways are shown in gray. All data were obtained from at least two batches of the enzyme, each measured in duplicate, to give at least four total measurements. The values are the average of the data obtained, while the error bars represent the standard deviation. *No rate acceleration was detected with this variant. †Data from Leveson-Gower et al., 2021.^17^ ‡Data from Mayer et al., 2019.^16^ Source data and measurement conditions provided in Tables S1 and S2.

Our initial efforts focused on the exploration of the mutational landscape of the two directed evolution campaigns by systematic evaluation of all possible single, double, and triple mutants from each lineage for both the apparent catalytic efficiency for the HyF reaction to form **3**, and yield and enantioselectivity for the FC reaction to give **6** (the selection parameters used during evolution, Figure 2). Overall, activity loss occurs far more easily than gain, where all three mutations are required to give the full benefit, however only one mutation was sufficient to produce nearly the whole activity loss (e.g., F93H causes a significant loss in activity for the FC reaction, as does S95G for the HyF reaction). This demonstrates that evolutionary pathways of this promiscuous designer enzyme (and previously demonstrated for non-natural reactivities in heme-enzymes^19,20^) closely reflect those found in nature. Loss of a nonselected activity occurring more rapidly than the gain of the selected activity is well-known for natural enzymes, and evidently also applies to designer enzymes–suggesting that evolutionary specialization might be an empirical property of enzyme catalysis, with relatively few exceptions.^2^ Only L18R proved mutually beneficial; although only slightly improving both reactivities, it can restore some hydrazone formation activity to the catalytically inactive S95G_M89N double mutant. Perhaps the most surprising finding is the strong epistatic interaction between the N19M and F93H mutations on hydrazone formation reactivity, which individually afford detrimental and mildly beneficial effects, respectively, yet, in combination, boost catalytic efficiency by an order of magnitude.

**Figure 3:**
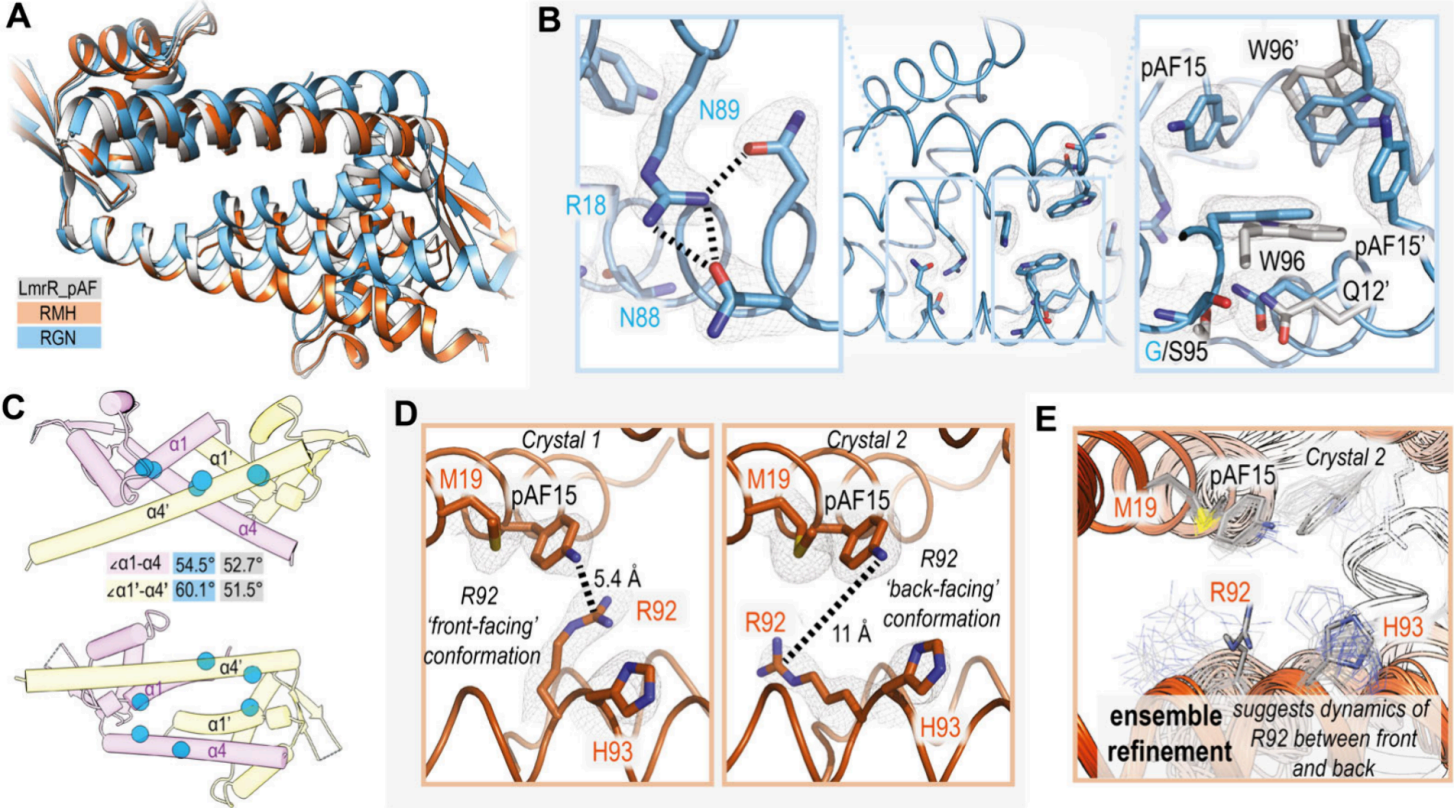
(A) Alignment of cartoon representation of the crystal structures of LmrR_pAF (gray, PDB: 6I8N) and the RGN mutant (blue) and RMH (orange) mutants. For clarity, a MOPS molecule bound in the pocket of LmrR_pAF is not shown. (B) Crystal structure of the RGN mutant with focus on the regions mutated, showing new hydrogen bonding interactions (left) and decrease in 96–96′ and 95–12′ distances compared to LmrR_pAF with alignment of the relevant residues shown in gray (PDB: 9GKT). (C) Cartoon depiction of the RGN mutant crustal structure with the α-carbon atoms of the mutated residues shown as blue spheres, angles between the α1/α4 and α1′/α4′ helices for RGN mutant (blue) and LmrR_pAF parent (gray) given in degrees. (D) Close up of the active site of the RMH mutant in the two crystal structures obtained showing differing conformations of the R92 side chain in crystals 1 (PDB: 9GKR) and 2 (PDB: 9GKS) with 2F_O_–F_C_ maps shown contoured at 1σ (left and middle, respectively); distance measurements are between the pAF15 side chain nitrogen atom and the R92 guanidinium central carbon atom in each monomer. (E) Result of ensemble refinement for crystal 2 showing that the diffraction data are partially consistent with a “forward-facing” conformation for R92. Summary of the crystallographic statistics provided in Table S3.

We successfully obtained crystals for both the RGN and RMH mutants, with one crystal diffracting to a maximum resolution of 2.45 Å for the former mutant and two crystals diffracting at 2.55 and 2.24 Å for the latter. Comparing the structure of LmrR_pAF^16^ with the RGN mutant reveals an unexpected change in the quaternary structure. With only three mutations, the assembly of the monomers to form the homodimeric structure is markedly different, as seen in 3D-structural alignments (Figure 3A). This is also reflected in a closing of the hydrophobic pocket formed at the dimer interface, where the distance between the two closest atoms of the W96/W96′ side chains decreases from 6.3 Å in LmrR_pAF to 4.0 Å in the RGN mutant (Figure 3B.). Accordingly, the pocket volume of the RGN mutant (∼800 Å^3^), as estimated using PyVOL,^21^ is around half that of LmrR_pAF (∼1600 Å^3^, Figure S1). The newly installed R18 side chain forms hydrogen bonds with carbonyl moieties in the N88 and N89 side chains (Figure 3B, the latter was also introduced in directed evolution). In one monomer, the H-bonding partner of R18 is E7′ rather than N88/N89; this interaction also likely affects the dimer assembly and hence the pocket volume (Figure S2). These new interactions may “pin” together the α1 and α4 helices and thus provide the large increase in angle, from 51.5° and 52.7° for each monomer LmrR_pAF to 54.5° and 60.1° for each monomer in the RGN mutant, which in turn allows tighter packing of the two monomers to form the homodimer affording the resulting smaller pocket volume (Figure 3C, Figure S2). We also observed an increase in proximity between α1 and α4′ helices (i.e., between two monomers forming the homodimer), which may be caused by increased space afforded for the Q12 side chain by the S95G mutant in RGN, allowing for closer packing of these helixes and gives a decreased distance between the α-carbons of these residues by almost 1 Å compared to LmrR_pAF (Figure 3B). Notably, while the L18R and M89N mutations have direct interactions in the RGN crystal structure, the L18R_M89N double mutant had no significant improvement over the L18R single mutant (Figure 2), suggesting the S95G mutation may be also essential in permitting the overall structural change observed.

Both structures obtained for the RMH mutant show a high degree of overall similarity with the LmrR_pAF parent (Figure 3A), despite the presence of a bound MOPS molecule in the LmrR_pAF crystal structure, indicating that mutations, and not crystallographic ligands, influence the pocket volume. However, RMH has new side chains installed proximal to the pAF residue (Figure 3D). While in both crystal structures, the mutated M19 and H93 residues are close to the reactive amino moiety of the pAF residue, the R92 side chain conformer varies significantly. In crystal 2, the side chain points to the back of the pocket in both monomers, whereas in one monomer in crystal 1, it comes near the pAF-amino moiety (Figure 3D). An unmodeled patch of density close to the pAF residue in crystal 2 prompted us to employ ensemble refinement in case the single best solution found did not well represent the conformational ensemble of the protein structure.^22−24^ Often such refinement is conducted on diffraction data obtained at noncryogenic temperatures,^25^ however, due to the high degree of conformational plasticity of the LmrR scaffold we could find diverse solutions using this technique on our existing, cryogenic, data (Figure S3). This also consistent with previous molecular dynamics (MD) simulations^26,27^ which demonstrate the flexibility of LmrR, and particularly the R92 residue in RMH, which was previously observed to flip between front- and backward-facing conformations.^27^ When applied to the RMH crystal structures, the R92 and H93 side chains in crystal 2 both exhibit considerable flexibility with R92 also have some forward-facing conformers in one monomer, evidencing the ability to move in closer proximity to the pAF residue and thus, the reaction center (Figure 3E).

**Figure 4:**
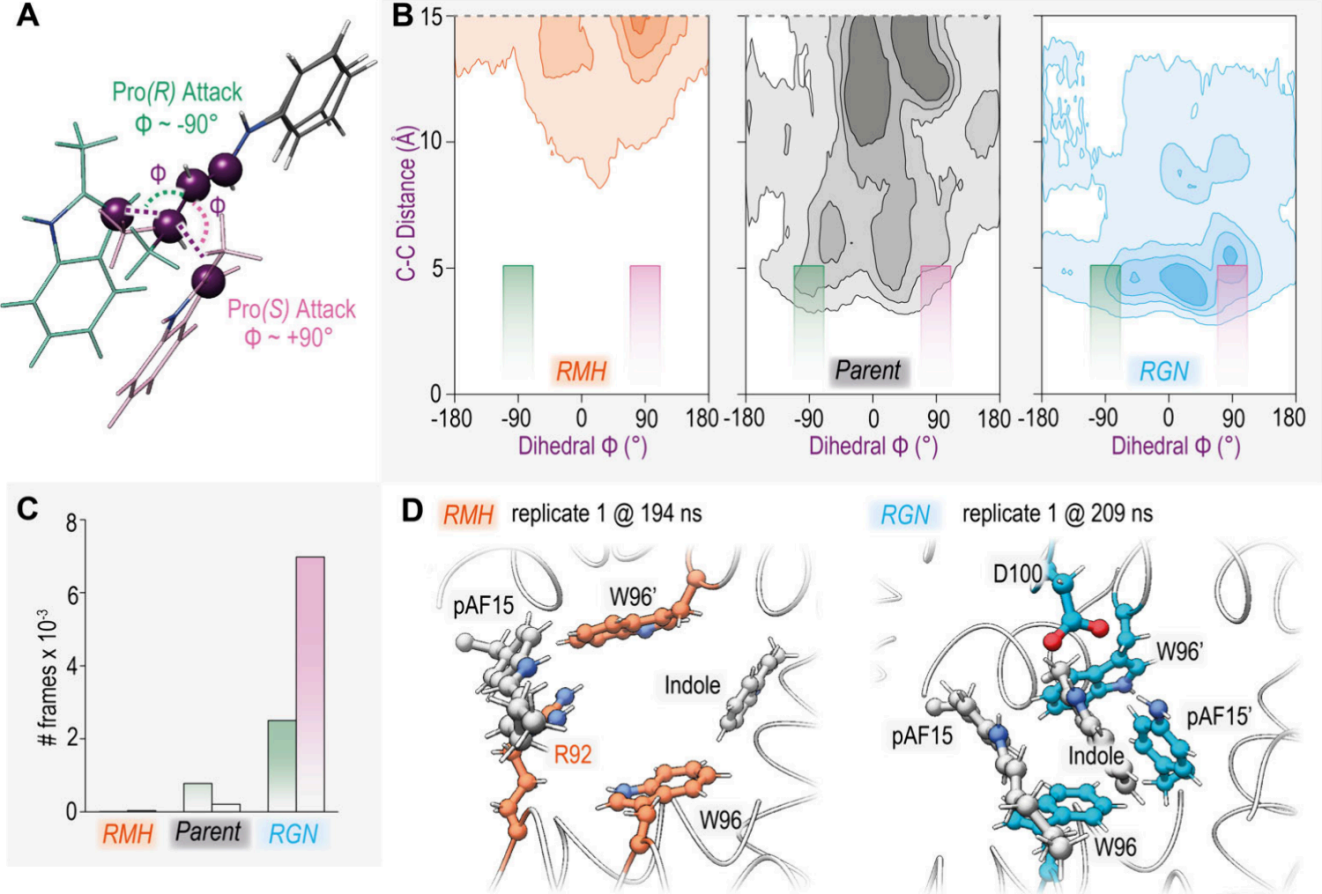
(A) Overlay of the transition states identified with a truncated model, showing how the dihedral between the four atoms shown in purple is diagnostic of the stereochemical outcome toward the (R) (green) and (S) (pink) enantiomers. (B) Measurements of the dihedral angle and reactive atom distance during MD simulations with the RMH (orange) LmrR_pAF parent (gray) and RGN (blue) variants, shown as a Gaussian kernel density estimate plot, where darker regions correspond to more dense populations. Total data obtained over 6 replicates of 500 ns (3 μs total per system). The green (left) and pink (right) boxes represent the cutoff values used for counting pro-(*R*) and pro-(*S*) NACs, respectively. Values over 15 Å are not shown—further details in Supporting Information. (C) NAC counts for the three studied variants, total obtained from all replicates (total frames = 3 × 10^5^). Pro-(*R*) shown in green (left) and pro-(*S*) in pink (right). (D) Representative frames of the top cluster based on analysis of the catalytic residue and 2-methyl-indole substrate for the RMH and RGN variants. These clusters represent 96.7% and 49.5% of the overall trajectory, respectively.

Using these experimentally determined structures as a basis, we proceeded with computational investigations to determine the molecular basis of the specialization phenomenon. Starting with the Friedel–Crafts reaction, we employed a multiscale methodology focusing on the identification of “near-attack conformers” (NACs) for the C–C bonding forming step. The NAC was defined based on the optimized structures of the transition state of the reactions resulting from DFT calculations with the B3LYP 6-31G(d,p) basis set, using a truncated reaction model of the reaction where pAF was substituted with aniline and hexenal was substituted with crotonaldehyde. The dihedral angle formed between the iminium, Cα, Cβ carbon atoms of the enal and C3 carbon atom of the indole substrate is diagnostic of the product stereochemistry with angles of ∼90° corresponding to the (*S*)-configured product, and those of ∼ −90° corresponding to the (*R*)-configured product (Figure 4A, Supporting Figure S4, Table S4). Using the same DFT B3LYP 6-31G(d,p) basis set, we optimized the structure of: ncAA pAF; the iminium species formed after condensation of the hexenal substrate at pAF; and 2-methylindole. Each component was then subjected to restrained electrostatic potential (RESP) calculations to obtain the charges, followed by parametrization with the GAFF force field using ambertools.^28^ These components were iteratively docked (first the iminium ion, then 2-methylindole) into the crystallographic structures using GOLD, and the top ranked solutions were chosen based on GOLDscore.^29^ Unrestrained MD simulations were performed for each LmrR_pAF and the RGN and RMH variants initiated from the geometries thus obtained to investigate the dynamics preceding the C–C bond forming step, with 6 replicates of 500 ns for each system. Convergence and stability of the trajectories were judged by stabilization of the RMSD (root-mean-square deviation) and PCA (principle component analysis). The distribution of NAC dihedral angles and C–C distances is shown as a density plot for each variant in Figure 4B (Figures S5–S7). In the RMH variant (orange), the indole substrate was barely maintained in the pocket, with an increase in substrate retention for the LmrR_pAF parent (gray), accurately reflecting the experimental reactivity trend between these two variants. Strikingly, in the RGN variant the 2-methyl-indole substrate remained in the pocket for five of the six replicates, with a clear bias toward dihedral angles indicative of the formation of the experimentally verified (*S*)-enantiomer^17^ upon close approaches (Figure 4C, Table S6). The correct enantioselectivity was not found with the LmrR_pAF parent, perhaps due to the lower intrinsic selectivity and lower sampling of near-attack conformations due to poorer ability to maintain the indole substrate in a productive conformation. This makes the fewer conformations of the close approaches of the substrates that do occur less significant for these simulations than with the RGN variants. Representative frames from the top clusters (using all atoms from the substrates for the analysis) from the MD simulations reveal that, upon close substrate approaches, the A92R mutation in the RMH variant occupies the space between the W96 and W96′ residues, which form the core of the hydrophobic pocket in LmrR (Figure 4D).

Conversely, in simulations with the RGN variant the tighter arrangement of W96/W96′ due to the smaller pocket volume makes an ideal binding pocket for the indole substrate, where the vicinal D100 residue could aid with the subsequent rearomatization process. Overall, the MD simulations suggest that while residues in RMH block the preferred substrate pocket for the Friedel–Crafts reaction to occur, the tighter pocket found in RGN favors productive substrate binding. Interestingly, all three studied variants maintain a similar overall flexibility as evidenced by RMSF calculations of the backbone (Figure S8). In RGN, the decreased pocket size promotes rigid substrate binding and preorganization for catalysis, but not a significant change in total protein dynamics.^30^ The indole substrate stays in the center of the hydrophobic pocket during MD simulations for the RGN variant, while rotameric control of the iminium ion ensures bias toward exposing the pro-(*S*) face for nucleophilic attack meaning that the overal enzyme-dynamics promote enantioselective catalysis.^31^

**Figure 5:**
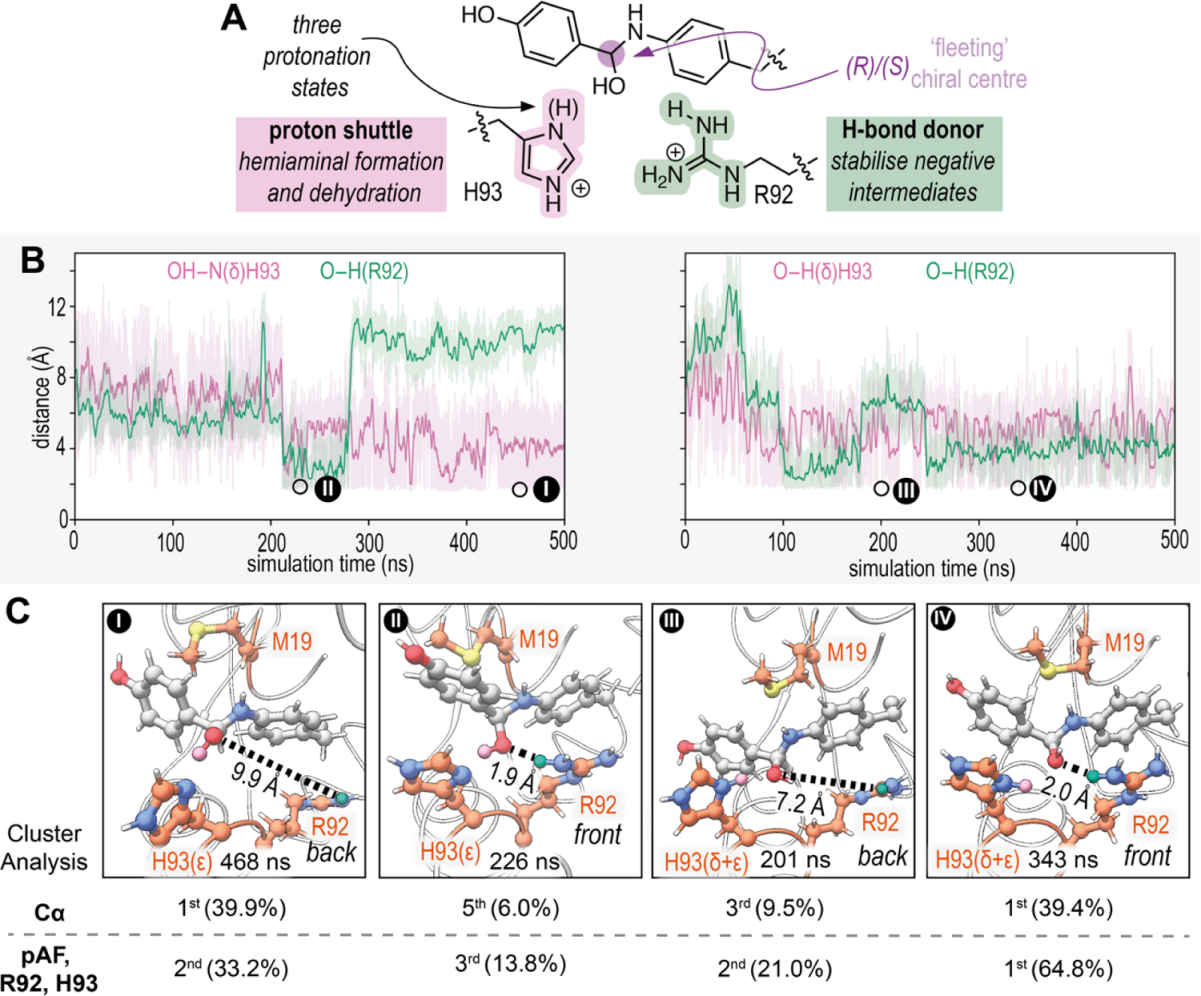
(A) Mechanistic hypothesis for the involvement of the R92 and H93 side chains in the RMH variant in hemiaminal formation and dehydration. (B) Distance measurements for the H-bonding interactions of the hemiaminal hydroxyl moiety and R92 (green) and H93 (pink) with the ε- (left) and doubly protonated states (right) and the (*S*)-configured hemiaminal intermediate, replicate numbers 3 and 2, respectively. (C) Snapshots of the simulations in (B) where the H93 and R92 residues participate in dual- or single-H-bonding interactions (front and back conformations of the dynamic R92 side chain). Estimated prevalence of these snapshots based on their occurrence in clusters analyzed using either the putitative catalytic residues (pAF15, R92, H93) or all C-alpha atoms. Distance measurements are between the R92 hydrogen atom and the hemiaminal oxygen atom. Full details are in the Supporting Information (Methods).

Next, we turned our attention to computational studies of the hydrazone formation reaction, where we focused on iminium ion formation between para-hydroxy benzaldehyde (**1**) and the pAF residue, which has previously been shown to be the rate-determining step when aniline-derivatives are used as catalyst.^32−36^ Thus, the simulations herein expand on our previous computational study of LmrR_pAF_RMH which considered only the substrate-free resting state rather than reaction intermediates with direct relevance to catalysis.^27^ We hypothesized that the R92 and H93 side chains introduced in the evolved RMH variant could assist with formation and dehydration of the hemiaminal intermediate by acting as a H-bond donor and proton-shuttle, respectively (Figure 5A). Together, these three residues could form semisynthetic catalytic machinery where they operate in a synergistic triad to boost catalysis. Following the same protocol as we applied for the modeling the FC reaction, we first performed MD simulations with the hemiaminal intermediate bound at one of the two pAF residues, permutating both the enantiomer of the fleeting chiral center of the hemiaminal and the protonation state of H93 to give a total of six distinct systems, conducting 500 ns simulations with 6 replicates for each system (Figures S9 to S14).^37^ In the ε-protonation state of H93, the δ-N atom makes persistent H-bond accepting interactions with the hemiaminal hydroxyl moiety, while interactions with R92 are only transient (Figure 5B, top). These interactions resemble a reaction intermediate after hemiaminal formation, suggesting that proton-transfer from δ-N of H93 could facilitate this process. However, participation of the R92 side chain by H-bonding is highly dynamic, with both backward- and forward-facing conformations present during the simulations, consistent with the crystallographic studies (Figure 5C). When H93 is doubly protonated, instead, the δ-H atom makes persistent H-bond donating interactions with the hemiaminal hydroxyl moiety, while interactions with R92 are once again highly dynamic (Figure 5B). These interactions could promote dehydration of the hemiaminal to form the iminium ion, again by proton transfer from H93 and potential participation of R92 by H-bonding (Figure 5C). We found that cation-π interactions between pAF and R92 are prominent in these simulations, however simultaneous cation-π and H-bonding interactions are not geometrically feasible (Figure S21). This suggests that cation-π interactions may be involved in biasing conformations of the R92 residue toward the front. Since electron-withdrawing substituents on aniline catalysts for hydrazone formation have been shown to be detrimental for catalytic rate while electron-donating substituents are beneficial, cation-π interactions are unlikely to play a direct role in the catalytic step.^38−40^

When we performed the corresponding MD simulations of the hemiaminal intermediate with the LmrR_pAF parent and RGN variants, we found that the hemiaminal intermediate makes nonproductive H-bonding interactions with a variety of other nonionizable amino acid side chains which cannot assist in proton transfer (Table S7). In the RGN variant, H-bonding to D100 is particularly persistent. This interaction would lead to reversion of the hemiaminal intermediate via deprotonation by the aspartate residue. In LmrR_pAF, several replicates show H-bonding interactions with N19, which explains the epistatic role of the N19M mutation in RMH – mutation to a hydrophobic residue prevents nonproductive interactions but does not directly promote catalysis in the absence of the H93 residue. Correspondingly, in the presence of the N19 residue, nonproductive H-bonding interactions persist, reducing the efficacy of H93 for proton transfer and resulting in the strong epistasis of the N19M and F93H mutations.

**Figure 6:**
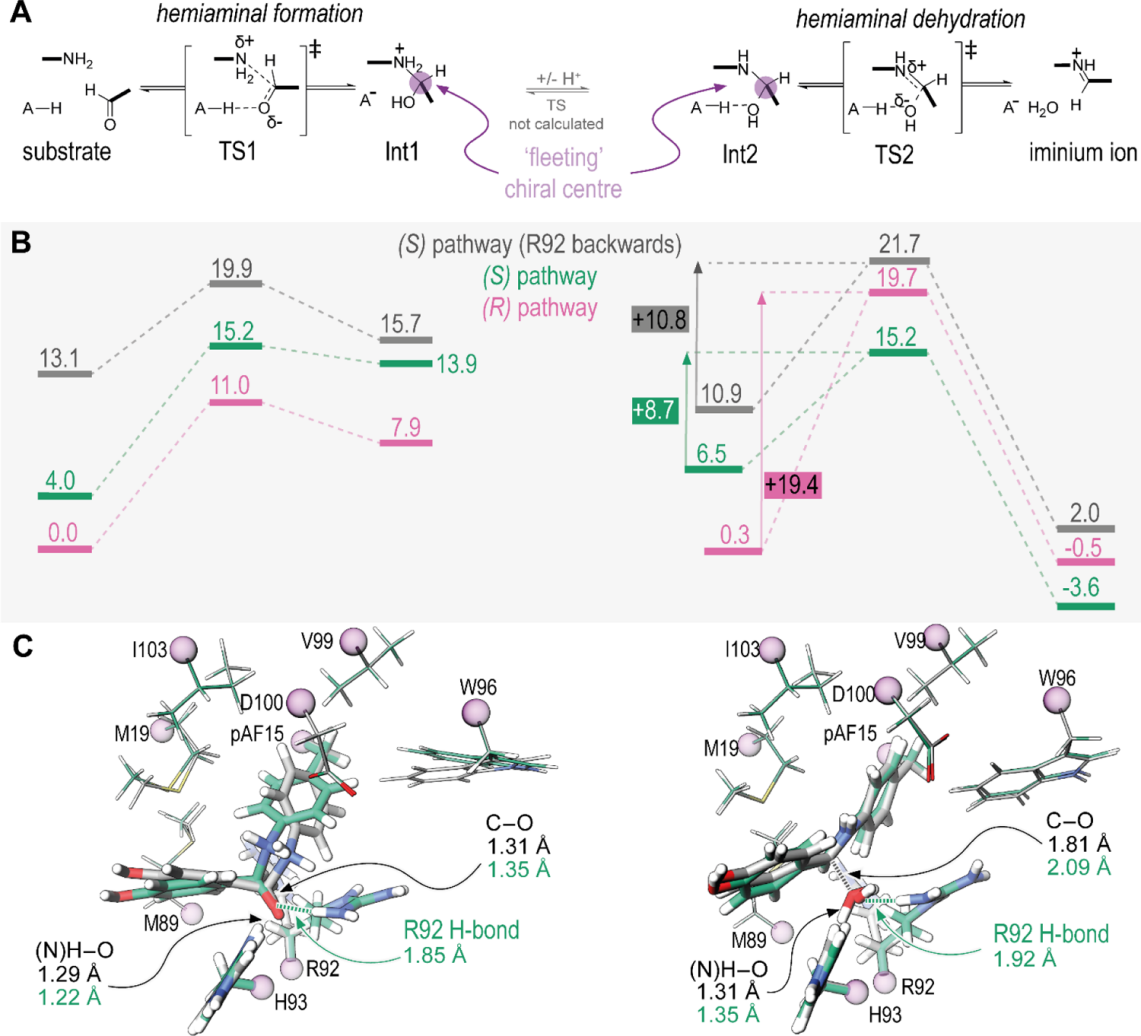
(A) Mechanism investigated for iminium formation via a hemiaminal intermediate assist by proton transfer from a specific acid residue (i.e., H93). (B) Energies along the reaction pathway for the intermediates and transition states listed in (A). Structures were optimized with the B3LYP 6-31G(d,p) functional, and the energies were recalculated with the B3LYP 6-311G++(2d,2p) functional. Values are given in kcal/mol. (C) Overlayed structures of the hemiaminal formation and dehydration transition states where R92 is in the forward or backward conformations (green and gray, respectively) with key bond lengths given. Frozen methyl groups are represented as purple spheres. Full details are in the Supporting Information.

Next, we applied the quantum cluster approach to determine how the interactions identified in the MD simulations affect the reaction barrier.^41,42^ We arbitrarily selected all residues with an atom within 6 Å of the pAF side chain nitrogen atom according to their crystallographic coordinates. We replaced backbone atoms with methyl groups, whose coordinates were fixed to produce a cluster with 165 atoms comprising the first shell of the catalytic sphere (Figures S15–S20). We used DFT to locate transition states and intermediates for a reaction pathway where H93 acts as a general-acid residue to promote hemiaminal formation and dehydration (Figure 6A).^43^ With the participation of both H93 and R92 (i.e., the front-facing conformer), the reaction proceeds preferentially via the (*S*)-configured hemiaminal, with the highest overall barrier for this reaction sequence being the hemiaminal formation 15.2 kcal/mol above the lowest energy substrate conformer, and the dehydration step proceeding with a barrier of 8.7 kcal/mol (Figure 6B – green). Interestingly, for the pathway via the (*R*)-configured hemiaminal, the hemiaminal formation proceeds with a lower barrier of 11.0 kcal/mol, but the dehydration provides a higher barrier of 19.4 kcal/mol, suggesting that this fleeting-chiral center has a significant implication for the overall reaction pathway, strongly disfavoring the (*R*)-configured pathway (Figure 6B, pink).

We then placed the R92 side chain in the backward-facing conformation and recalculated the energy profile for the (*S*)-configured pathway, finding that the barrier for dehydration increased by 2.1 kcal/mol. This difference, while relatively small, suggests that the forward-facing R92 conformer may play a role in catalysis. This energy difference corresponds to approximately a 30-fold rate acceleration, while the real contribution of the R92 side-chain is around 5-fold, consistent with dynamics between forward- and backward- conformations reducing the efficacy of this component of the catalytic machinery. Noncovalent interaction (NCI) analysis on the transition state structures was also conducted for both the forward- and backward-facing R92 conformations in the (*S*)-configured pathway to identify which NCIs may explain the role of R92 in lowering the transition-state energy (Figure S22).^44^ This analysis identified a hydrogen-bonding interaction between R92 and the hemiaminal-oxo moiety in the forward-facing conformation, which is not present in the backward-facing pathway. This interaction is likely the reason for the lower barrier since the hydrogen-bonding stabilizes the negatively charged intermediates. After constructing a cluster from equivalent atoms taking the crystallographic positions found in the other monomer of RMH crystal 2 (which also has a backward-facing R92 conformation), we found only 0.1 kcal/mol difference in barrier for hemiaminal dehydrations compared to the backward facing conformation produced from the other monomer (Figure S15 and S23). This clear agreement between the clusters suggests that the 2.1 kcal/mol energy-barrier difference based on the R92 conformers is not an artifact of small movements of other moieties in the quantum cluster. The effect of the forward-facing R92 conformation is also apparent from inspection of transition state structures, where H-bonding to the oxo/hydroxyl moiety from the aldehyde substrate results in “later” transition states with greater degrees of proton transfer and incipient bond formation compared to the backward-facing R92 conformation (Figure 6C). Our previous MD studies of the RMH-variant substrate-free resting state demonstrated that the motion of the two R92 side chains in each monomer is sensitive to mutations, with the cis-forward conformation (i.e., both R92 and R92’ facing toward the respective pAF15 residues) being greatly diminished in improved mutants.^27^ The studies herein suggest that the trans-conformation (i.e., R92 forward and R92’ backward, as observed in RMH crystal 1) may be the catalytically active conformation.

## Conclusion

Overall, our study probed the divergent evolutionary pathways of a promiscuous designer enzyme by systematic evaluation of each mutant along both lineages revealing a rapid specialization effect and epistasis.^45^ Crystallographic structural elucidation of the evolved mutants, along with our previous structure of the parent, revealed stark differences in the manner of adaptation of the protein scaffold for its selected task. For the Friedel–Crafts reaction the evolved RGN mutant exhibits a reaction pocket decreased in size, due to a marked change in quarternary structure and a closing of the dimer interface that forms the pocket. MD simulations evidenced more rigid and preorganized substrate binding as well as a bias toward the preferred stereochemical pathway.^29,41^ The structural effect of the RGN mutations was largely due to their occurrence in the so-called “hinge” region of LmrR (where the end of helix α1 meets the beginning of helix α4), where we previously also saw that incorporation of a boron-based ncAA significantly changed the overall structure of LmrR.^27,46,47^ This suggests that this region should be further targeted in future enzyme design and engineering campaigns based on the popular LmrR scaffold. Conversely, the evolved mutant for the hydrazone formation reaction maintains an overall structure highly similar to the LmrR_pAF parent, with a few key functional side chains introduced into the primary reaction sphere, which make persistent or transient interactions with the key reaction intermediate (hemiaminal) during MD simulations. Using a quantum cluster approach, we could show how the catalytic ncAA and two other side chains introduced in directed evolution form a semisynthetic catalytic machine for efficient catalysis.^48^ Directed evolution produced a catalytic solution for iminium ion formation employing a histidine residue for proton transfer, distinct from Class I Aldolases that employ a water molecule coordinated by a glutamate or tyrosine side chain.^49,50^ Further development of designer enzymes that employ iminium catalysis can now benefit from this proven motif in design and engineering efforts. With increased understanding of the molecular phenomena underlying designer enzymes’ catalytic specialization, the programmable construction of highly active designer enzymes for a given task will be more readily realized.

## Data Availability

Crystal structures of LmrR_pAF_RMH (crystals 1 and 2) and LmrR_pAF_RGN have been deposited in the Protein Data Bank with accession codes 9GKR, 9GKS, and 9GKT, respectively.

## Supporting Information Available

Tables S1–S7 and Figures S1–S20, along with the Experimental and Computational Methods (PDF)
Geometries of stationary points and the transition states are provided in a separate file as Cartesian coordinates, along with absolute calculated energies in Hartrees (TXT)

## References

[ref1] Khersonsky O.; Tawfik D. S. Enzyme Promiscuity: A Mechanistic and Evolutionary Perspective. Annu. Rev. Biochem. 2010, 79 (1), 471–505. 10.1146/annurev-biochem-030409-143718. 20235827

[ref2] Soskine M.; Tawfik D. S. Mutational Effects and the Evolution of New Protein Functions. Nat. Rev. Genet. 2010, 11 (8), 572–582. 10.1038/nrg2808. 20634811

[ref3] Davidi D.; Longo L. M.; Jabłońska J.; Milo R.; Tawfik D. S. A Bird’s-Eye View of Enzyme Evolution: Chemical, Physicochemical, and Physiological Considerations. Chem. Rev. 2018, 118 (18), 8786–8797. 10.1021/acs.chemrev.8b00039. 30133258

[ref4] Jensen R. A. Enzyme Recruitment in Evolution of New Function. Annu. Rev. Microbiol. 1976, 30 (1), 409–425. 10.1146/annurev.mi.30.100176.002205. 791073

[ref5] Yčas M. On Earlier States of the Biochemical System. J. Theor. Biol. 1974, 44 (1), 145–160. 10.1016/S0022-5193(74)80035-4. 4207200

[ref6] Bornscheuer U. T.; Kazlauskas R. J. Catalytic Promiscuity in Biocatalysis: Using Old Enzymes to Form New Bonds and Follow New Pathways. Angew. Chem., Int. Ed. 2004, 43 (45), 6032–6040. 10.1002/anie.200460416. 15523680

[ref7] Leveson-Gower R. B.; Mayer C.; Roelfes G. The Importance of Catalytic Promiscuity for Enzyme Design and Evolution. Nat. Rev. Chem. 2019, 3 (12), 687–705. 10.1038/s41570-019-0143-x.

[ref8] Toscano M. D.; Woycechowsky K. J.; Hilvert D. Minimalist Active-Site Redesign: Teaching Old Enzymes New Tricks. Angew. Chem., Int. Ed. 2007, 46 (18), 3212–3236. 10.1002/anie.200604205. 17450624

[ref9] Sheldon R. A.; Woodley J. M. Role of Biocatalysis in Sustainable Chemistry. Chem. Rev. 2018, 118 (2), 801–838. 10.1021/acs.chemrev.7b00203. 28876904

[ref10] Sheldon R. A.; Brady D.; Bode M. L. The Hitchhiker’s Guide to Biocatalysis: Recent Advances in the Use of Enzymes in Organic Synthesis. Chem. Sci. 2020, 11 (10), 2587–2605. 10.1039/C9SC05746C. 32206264 PMC7069372

[ref11] Schwizer F.; Okamoto Y.; Heinisch T.; Gu Y.; Pellizzoni M. M.; Lebrun V.; Reuter R.; Köhler V.; Lewis J. C.; Ward T. R. Artificial Metalloenzymes: Reaction Scope and Optimization Strategies. Chem. Rev. 2018, 118 (1), 142–231. 10.1021/acs.chemrev.7b00014. 28714313

[ref12] Chen K.; Arnold F. H. Engineering New Catalytic Activities in Enzymes. Nat. Catal. 2020, 3 (3), 203–213. 10.1038/s41929-019-0385-5.

[ref13] Romero E.; Jones B. S.; Hogg B. N.; Rué Casamajo A.; Hayes M. A.; Flitsch S. L.; Turner N. J.; Schnepel C. Enzymatic Late-Stage Modifications: Better Late Than Never. Angew. Chem., Int. Ed. 2021, 60 (31), 16824–16855. 10.1002/anie.202014931. PMC8359417 33453143

[ref14] Drienovská I.; Mayer C.; Dulson C.; Roelfes G. A Designer Enzyme for Hydrazone and Oxime Formation Featuring an Unnatural Catalytic Aniline Residue. Nat. Chem. 2018, 10 (9), 946–952. 10.1038/s41557-018-0082-z. 29967395

[ref15] Erkkilä A.; Majander I.; Pihko P. M. Iminium Catalysis. Chem. Rev. 2007, 107 (12), 5416–5470. 10.1021/cr068388p. 18072802

[ref16] Mayer C.; Dulson C.; Reddem E.; Thunnissen A.-M. W. H.; Roelfes G. Directed Evolution of a Designer Enzyme Featuring an Unnatural Catalytic Amino Acid. Angew. Chem., Int. Ed. 2019, 58 (7), 2083–2087. 10.1002/anie.201813499. PMC6519144 30575260

[ref17] Leveson-Gower R. B.; Zhou Z.; Drienovská I.; Roelfes G. Unlocking Iminium Catalysis in Artificial Enzymes to Create a Friedel-Crafts Alkylase. ACS Catal. 2021, 11 (12), 6763–6770. 10.1021/acscatal.1c00996. 34168902 PMC8218303

[ref18] Leveson-Gower R. B.; de Boer R. M.; Roelfes G. Tandem Friedel-Crafts-Alkylation-Enantioselective-Protonation by Artificial Enzyme Iminium Catalysis. ChemCatChem. 2022, 14 (8), e202101875 10.1002/cctc.202101875. 35915643 PMC9313897

[ref19] Kan S. B. J.; Lewis R. D.; Chen K.; Arnold F. H. Directed Evolution of Cytochrome c for Carbon-Silicon Bond Formation: Bringing Silicon to Life. Science 2016, 354 (6315), 1048–1051. 10.1126/science.aah6219. 27885032 PMC5243118

[ref20] Garcia-Borràs M.; Kan S. B. J.; Lewis R. D.; Tang A.; Jimenez-Osés G.; Arnold F. H.; Houk K. N. Origin and Control of Chemoselectivity in Cytochrome c Catalyzed Carbene Transfer into Si-H and N-H Bonds. J. Am. Chem. Soc. 2021, 143 (18), 7114–7123. 10.1021/jacs.1c02146. 33909977 PMC9292473

[ref21] Smith R. H. B.; Dar A. C.; Schlessinger A. PyVOL: A PyMOL Plugin for Visualization, Comparison, and Vol. Calculation of Drug-Binding Sites. bioRxiv Preprint, October 24, 2019. 10.1101/816702.

[ref22] Levin E. J.; Kondrashov D. A.; Wesenberg G. E.; Phillips G. N. Ensemble Refinement of Protein Crystal Structures: Validation and Application. Struct. London Engl. 1993 2007, 15 (9), 1040–1052. 10.1016/j.str.2007.06.019. PMC2039884 17850744

[ref23] Burnley B. T.; Afonine P. V.; Adams P. D.; Gros P. Modelling Dynamics in Protein Crystal Structures by Ensemble Refinement. eLife 2012, 1, e00311 10.7554/eLife.00311. 23251785 PMC3524795

[ref24] Broom A.; Rakotoharisoa R. V.; Thompson M. C.; Zarifi N.; Nguyen E.; Mukhametzhanov N.; Liu L.; Fraser J. S.; Chica R. A. Ensemble-Based Enzyme Design Can Recapitulate the Effects of Laboratory Directed Evolution in Silico. Nat. Commun. 2020, 11 (1), 4808 10.1038/s41467-020-18619-x. 32968058 PMC7511930

[ref25] Fraser J. S.; van den Bedem H.; Samelson A. J.; Lang P. T.; Holton J. M.; Echols N.; Alber T. Accessing Protein Conformational Ensembles Using Room-Temperature X-Ray Crystallography. Proc. Natl. Acad. Sci. U. S. A. 2011, 108 (39), 16247–16252. 10.1073/pnas.1111325108. 21918110 PMC3182744

[ref26] Alonso-Cotchico L.; Rodríguez-Guerra Pedregal J.; Lledós A.; Maréchal J.-D. The Effect of Cofactor Binding on the Conformational Plasticity of the Biological Receptors in Artificial Metalloenzymes: The Case Study of LmrR. Front. Chem. 2019, 7, na 10.3389/fchem.2019.00211. PMC6467942 31024897

[ref27] Casilli F.; Canyelles-Niño M.; Roelfes G.; Alonso-Cotchico L. Computation-Guided Engineering of Distal Mutations in an Artificial Enzyme. Faraday Discuss. 2024, 252, 262 10.1039/D4FD00069B. 38836699 PMC11389854

[ref28] Case D. A.; Aktulga H. M.; Belfon K.; Cerutti D. S.; Cisneros G. A.; Cruzeiro V. W. D.; Forouzesh N.; Giese T. J.; Götz A. W.; Gohlke H.; Izadi S.; Kasavajhala K.; Kaymak M. C.; King E.; Kurtzman T.; Lee T.-S.; Li P.; Liu J.; Luchko T.; Luo R.; Manathunga M.; Machado M. R.; Nguyen H. M.; O’Hearn K. A.; Onufriev A. V.; Pan F.; Pantano S.; Qi R.; Rahnamoun A.; Risheh A.; Schott-Verdugo S.; Shajan A.; Swails J.; Wang J.; Wei H.; Wu X.; Wu Y.; Zhang S.; Zhao S.; Zhu Q.; Cheatham T. E. I.; Roe D. R.; Roitberg A.; Simmerling C.; York D. M.; Nagan M. C.; Merz K. M. Jr. AmberTools. J. Chem. Inf. Model. 2023, 63 (20), 6183–6191. 10.1021/acs.jcim.3c01153. 37805934 PMC10598796

[ref29] Verdonk M. L.; Cole J. C.; Hartshorn M. J.; Murray C. W.; Taylor R. D. Improved Protein-Ligand Docking Using GOLD. Proteins Struct. Funct. Bioinforma. 2003, 52 (4), 609–623. 10.1002/prot.10465. 12910460

[ref30] Preiswerk N.; Beck T.; Schulz J. D.; Milovník P.; Mayer C.; Siegel J. B.; Baker D.; Hilvert D. Impact of Scaffold Rigidity on the Design and Evolution of an Artificial Diels-Alderase. Proc. Natl. Acad. Sci. U. S. A. 2014, 111 (22), 8013–8018. 10.1073/pnas.1401073111. 24847076 PMC4050586

[ref31] Glowacki D. R.; Harvey J. N.; Mulholland A. J. Taking Ockham’s Razor to Enzyme Dynamics and Catalysis. Nat. Chem. 2012, 4 (3), 169–176. 10.1038/nchem.1244. 22354430

[ref32] Larsen D.; Pittelkow M.; Karmakar S.; Kool E. T. New Organocatalyst Scaffolds with High Activity in Promoting Hydrazone and Oxime Formation at Neutral pH. Org. Lett. 2015, 17 (2), 274–277. 10.1021/ol503372j. 25545888 PMC4301078

[ref33] Cordes E. H.; Jencks W. P. Nucleophilic Catalysis of Semicarbazone Formation by Anilines. J. Am. Chem. Soc. 1962, 84 (5), 826–831. 10.1021/ja00864a030.

[ref34] Cordes E. H.; Jencks W. P. On the Mechanism of Schiff Base Formation and Hydrolysis. J. Am. Chem. Soc. 1962, 84 (5), 832–837. 10.1021/ja00864a031.

[ref35] Dirksen A.; Dirksen S.; Hackeng T. M.; Dawson P. E. Nucleophilic Catalysis of Hydrazone Formation and Transimination: Implications for Dynamic Covalent Chemistry. J. Am. Chem. Soc. 2006, 128 (49), 15602–15603. 10.1021/ja067189k. 17147365

[ref36] Thygesen M. B.; Munch H.; Sauer J.; Cló E.; Jørgensen M. R.; Hindsgaul O.; Jensen K. J. Nucleophilic Catalysis of Carbohydrate Oxime Formation by Anilines. J. Org. Chem. 2010, 75 (5), 1752–1755. 10.1021/jo902425v. 20131837

[ref37] Reetz M. T.; Garcia-Borràs M. The Unexplored Importance of Fleeting Chiral Intermediates in Enzyme-Catalyzed Reactions. J. Am. Chem. Soc. 2021, 143 (37), 14939–14950. 10.1021/jacs.1c04551. 34491742 PMC8461649

[ref38] Canal-Martín A.; Navo C. D.; Sáez E.; Molero D.; Jiménez-Osés G.; Pérez-Fernández R. Nucleophilic Catalysis of *p* -Substituted Aniline Derivatives in Acylhydrazone Formation and Exchange. Org. Biomol. Chem. 2021, 19 (33), 7202–7210. 10.1039/D1OB00871D. 34612342

[ref39] Yuen L. H.; Saxena N. S.; Park H. S.; Weinberg K.; Kool E. T. Dark Hydrazone Fluorescence Labeling Agents Enable Imaging of Cellular Aldehydic Load. ACS Chem. Biol. 2016, 11 (8), 2312–2319. 10.1021/acschembio.6b00269. 27326450 PMC5503141

[ref40] Crisalli P.; Kool E. T. Water-Soluble Organocatalysts for Hydrazone and Oxime Formation. J. Org. Chem. 2013, 78 (3), 1184–1189. 10.1021/jo302746p. 23289546 PMC3562402

[ref41] Himo F.; de Visser S. P. Status Report on the Quantum Chemical Cluster Approach for Modeling Enzyme Reactions. Commun. Chem. 2022, 5 (1), 1–4. 10.1038/s42004-022-00642-2. 36697758 PMC9814711

[ref42] Sheng X.; Himo F. The Quantum Chemical Cluster Approach in Biocatalysis. Acc. Chem. Res. 2023, 56 (8), 938–947. 10.1021/acs.accounts.2c00795. 36976880 PMC10116590

[ref43] Kirmizialtin S.; Yildiz B. S.; Yildiz I. A DFT-Based Mechanistic Study on the Formation of Oximes. J. Phys. Org. Chem. 2017, 30 (12), e3711 10.1002/poc.3711.

[ref44] Johnson E. R.; Keinan S.; Mori-Sánchez P.; Contreras-García J.; Cohen A. J.; Yang W. Revealing Noncovalent Interactions. J. Am. Chem. Soc. 2010, 132 (18), 6498–6506. 10.1021/ja100936w. 20394428 PMC2864795

[ref45] Miton C. M.; Campbell E. C.; Kaczmarski J. A.; Feixas F.; Romero-Rivera A.; Sandhu M.; Anderson D. W.; Shatani N.; Osuna S.; Jackson C. J.; Tokuriki N. Origin of Evolutionary Bifurcation in an Enzyme. bioRxiv Preprint, November 26, 2023. 10.1101/2023.11.25.568631.

[ref46] Longwitz L.; Leveson-Gower R. B.; Rozeboom H. J.; Thunnissen A.-M. W. H.; Roelfes G. Boron Catalysis in a Designer Enzyme. Nature 2024, 629 (8013), 824–829. 10.1038/s41586-024-07391-3. 38720081

[ref47] Takeuchi K.; Tokunaga Y.; Imai M.; Takahashi H.; Shimada I. Dynamic Multidrug Recognition by Multidrug Transcriptional Repressor LmrR. Sci. Rep. 2014, 4 (1), 6922 10.1038/srep06922. 25403615 PMC4235314

[ref48] Obexer R.; Godina A.; Garrabou X.; Mittl P. R. E.; Baker D.; Griffiths A. D.; Hilvert D. Emergence of a Catalytic Tetrad during Evolution of a Highly Active Artificial Aldolase. Nat. Chem. 2017, 9 (1), 50–56. 10.1038/nchem.2596. 27995916

[ref49] Heine A.; DeSantis G.; Luz J. G.; Mitchell M.; Wong C.-H.; Wilson I. A. Observation of Covalent Intermediates in an Enzyme Mechanism at Atomic Resolution. Science 2001, 294 (5541), 369–374. 10.1126/science.1063601. 11598300

[ref50] Schneider S.; Sandalova T.; Schneider G.; Sprenger G. A.; Samland A. K. Replacement of a Phenylalanine by a Tyrosine in the Active Site Confers Fructose-6-Phosphate Aldolase Activity to the Transaldolase of Escherichia Coli and Human Origin. J. Biol. Chem. 2008, 283 (44), 30064–30072. 10.1074/jbc.M803184200. 18687684 PMC2662071

