# Supplementary material for: Evolutionary Specialization of a Promiscuous Designer Enzyme
Source: ACS Catal. 2025 Jan 13;15(3):1544–52. doi: 10.1021/acscatal.4c06409 (PMC11811923; doi:10.1021/acscatal.4c06409)
Supplement: Supplementary file 1 — cs4c06409_si_001.pdf [file cs4c06409_si_001.pdf]

Supporting Information

Evolutionary Specialization of a Promiscuous Designer Enzyme

Reuben B. Leveson-Gower[1,4],* Laura Tiessler-Sala[2], Henriette J. Rozeboom[3], Andy-Mark W.H. Thunnissen[3], Jean-Didier Maréchal[2], Gerard Roelfes[1]*

[1] Stratingh Institute for Chemistry, University of Groningen, 9747AG Groningen, The Netherlands

[2] Insilichem, Departament de Química, Universitat Autònoma de Barcelona, 08193 Cerdanyola del Vallès, Spain

[3] Groningen Biomolecular Sciences and Biotechnology Institute, University of Groningen, 9747AG Groningen, The Netherlands

[4] Present address: Biocatalysis section, Department of Biotechnology, Delft University of Technology, van der Maasweg 9, 2629HZ Delft, The Netherlands

*To whom correspondence should be addressed: r.leveson-gower@tudelft.nl; j.g.roelfes@rug.nl

Contents

Supporting Tables

Table S1: Catalytic Efficiencies; Hydrazone Formation S3

Table S2: Yield and Enantioselectivity; Friedel-Crafts Reaction S4

Table S3: Crystallographic Statistics S5

Table S4: Friedel-Crafts TS Features S6

Table S5: NAC Counts Friedel-Crafts Reaction S8

Table S6-S7: H-Bond Analysis of Hemiaminal MD Simulations S8

Supporting Figures

Figure S1: Pocket volume calculations S12

Figure S2: Structural Changes in the RGN Mutant S13

Figure S3: Ensemble Refinement Structures S14

Figure S4: Transition States for NAC Definition Friedel-Crafts Reaction S15

Figures S5-S7: Time course plots for Friedel-Crafts MD Simulations S16

Figure S7: RMSF Plots from Friedel-Crafts MD Simulations S19

Figures S9-S14: Time course plots for Hemiaminal MD Simulations S20

Figure S15: Energy Level Diagram for QM Cluster Models S26

Figures S16-S20: Structures of Intermediates and Transition States for QM Cluster Models S27

Experimental Methods S32

Computational Methods S37

References S39

Supporting Tables

Table S1: Apparently catalytic efficiencies for the mutants studied herein for the hydrazone formation reaction, determined as described below. Catalysis was not observed for LmrR_pAF_S95G_M89N at 5 µM.

Mutant	$(K_{cat}/K_M)_{app}$ $M^{-1}s^{-1}$	S.D.
LmrR_pAF_A92R_N19M_F93H[1]	103.0	5.0
LmrR_pAF_N19M_F93H	18.6	2.1
LmrR_pAF_A92R_N19M[1]	12.0	2.6
LmrR_pAF_A92R_F93H[1]	6.1	1.3
LmrR_pAF _F93H	2.3	0.3
LmrR_pAF _N19M	0.6	0.1
LmrR_pAF_A92R[1]	4.0	1.0
LmrR_pAF[1]	1.9	0.1
LmrR_pAF_L18R	2.3	0.5
LmrR_pAF_S95G	0.6	0.1
LmrR_pAF_M89N	0.7	0.1
LmrR_pAF_L18R_S95G	0.5	0.1
LmrR_pAF_L18R_M89N	2.1	0.1
LmrR_pAF_S95G_M89N	N.D.	
LmrR_pAF_L18R_S95G_M89N	0.6	0.2

Conducted as previously described[1]. Kinetic assays for the hydrazone formation between 4-HBA and NBD-H were performed following product formation at 472 nm (ε_{472} = 25,985 M−1 cm^{-1}). All measurements were at 25 °C in buffer A containing 5% (v/v) DMF, 4-HBA (5 mM) and NBD-H (50 µM) enzyme concentrations (with respect to the dimer) ranged from 2 µM to 5 µM depending on the activity of the LmrR_pAF variant. Background rates were recorded for each measurement (10 – 30 minutes) before addition of the enzyme. Initial velocities were corrected for the background rate and used to calculated the apparent catalytic efficiency.

Table S2: Yield and enantioselectivity for the Friedel-Crafts reaction, determined as described below.

Mutant	Yield 6 (%)	S.D.	e.e. 6 (%)	S.D.
LmrR_pAF_A92R_N19M_F93H*	15	1.2	59	1.7
LmrR_pAF_A92R_N19M_F93H	8	1.5	60	2.3
LmrR_pAF_N19M_F93H	11	0.6	53	0.3
LmrR_pAF_A92R_N19M	26	3.1	46	2.4
LmrR_pAF_A92R_F93H	4	0.2	44	0.3
LmrR_pAF _F93H	8	0.4	46	1.2
LmrR_pAF _N19M	25	0.6	36	0.4
LmrR_pAF_A92R	18	4.5	51	0.7
LmrR_pAF[2]	42	4.4	45	0.3
LmrR_pAF_L18R[2]	58	2.0	67	0.2
LmrR_pAF_S95G[2]	46	7.6	55	0.7
LmrR_pAF_M89N	35	0.9	51	0.4
LmrR_pAF_L18R_S95G[2]	70	8.1	78	0.3
LmrR_pAF_L18R_M89N	49	0.9	73	0.3
LmrR_pAF_S95G_M89N	23	1.6	60	0.2
LmrR_pAF_L18R_S95G_M89N[2]	73	2.8	87	0.7
LmrR_pAF_L18R_S95G_M89N*	82	8.0	90	0.2

Conducted as previously described[2]. Reactions were conducted in 300 µL total volume in a 2 mL microcentrifuge tube. Stock solutions of protein buffer (150 mM NaCl, 50 mM NaH_2PO_4 pH = 6.5) to give the specified final concentration and the same buffer was added to make up 276 µL volume. Stock solutions of indole (25 mM in DMF, 12 µL added, final concentration 1 mM) and trans-2-hexenal (125 mM, 12 µL added to give final a concentration of 5 mM) substrates were added. The microcentrifuge tubes were then mixed by continuous inversion in a cold room as 4 °C for 16 hours. After the reaction time had elapsed, $NaBH_4$ solution (60 µL, 20 mg/mL in 0.5 w/v % NaOH) and 3-(3-hydroxypropylindole) internal standard solution (12 µL, 5 mM in DMF) were added. The micro-centrifuge tubes were mixed by continuous inversion for a further 30 minutes. The reaction products and internal standard were then extracted by vortex mixing with EtOAc (1 mL) and the organic extract was dried over Na_2SO_4, filtered and evaporated to dryness. The residue thus obtained was redissolved by vortex mixing with HPLC grade solvent (heptane:isopropanol 4:1, 90 µL) and analysed by normal phase HPLC using a Chiralcel OD-H column (heptane:isopropanol 80:20, 1 mL/min) to determine yield and enantioselectivity with a 20 µL injection volume according to a previously prepared calibration curve. *Contains the D55K, Q59K mutations used for crystallisation purposes, results are from a triplicate experiment with a single protein batch. In all other cases the results are the average of two experiments with different batches of protein, each conducted in duplicate, to give at least four total data points.

Table S3. Summary of the crystallographic statistics for the LmrR variants. *All variants contain the additional mutations D55K Q59K which reverts the DNA binding abrogating mutations, but promotes the formation of well diffracting crystals, and does not significantly affect catalytic activity (Table S2)[3].

	**RMH*-1**	**RMH*-2**	**RGN***
Data collection			
Beamline	ESRF/ID23-2	ESRF/ID30A-1	ESRF/ID30A-1
Wavelength (Å)	0.87313	0.96546	0.96546
Resolution (Å)	44 – 2.55<br>(2.66 – 2.55)	52 – 2.24<br>(2.31 – 2.24)	126 – 2.45<br>(2.55 – 2.45)
Space group	C2	C2	$P2_12_12_1$
Unit cell dimensions a,b,c (Å)	107.03, 36.46, 69.12<br>β=97.78°	104.93, 35.30, 68.18<br>β=97.27°	38.53, 49.96, 125.73
$CC_{(1/2)}$	0.996 (0.409)	0.999 (0.467)	0.997 (0.531)
<I/σ>	7.7 (1.0)	9.9 (1.0)	8.9 (1.5)
R_{merge}	0.137 (1.750)	0.042 (0.570)	0.121 (1.005)
R_{pim}	0.063 (0.788)	0.029 (0.403)	0.063 (0.525)
Completeness (%)	99.8 (99.9)	97.9 (86.2)	99.2 (97.0)
Multiplicity	5.7 (6.0)	2.9 (2.8)	4.3 (4.3)
Refinement			
R_{work} /R_{free} (%)	0.259/0.324	0.227/0.294	0.215/0.286
Model composition in AU:<br>polypeptide chains, amino acid residues, waters	A: aa 4-70, 73-115<br>B: aa 4-70, 75-110	A: aa 4-69, 75-111<br>B: aa 2-68, 75-111<br>22 waters	A: aa 4-68, 76-111<br>B: aa 4-68, 76-119<br>26 waters
Average B-factors, protein/solvent ($Å^2$)	78.3	82.6/58.8	55.7/43.0
RMSZ bond lengths/angles	0.236/0.404	0.635/0.741	0.356/0.519
Rama preferred/outliers (%)	100.0/0.0	99.0/0.0	99.5/0.0
Rotamers outliers (%)	1.1	1.6	0.6
Molprobity clashscore	6.5	7.2	7.7
PDB entry	9GKR	9GKS	9GKT

Table S4. Summary of geometrical features and energies of the transition states and respective reactants and products for the truncated model used for NAC definition for the Friedel-Crafts reaction. Geom. refers to the relative conformation of the iminium ion double bonds, dihedral refers to the dihedral between the carbonyl, C-*alpha*, C-*beta* and indole C-3 atoms. Distance refers to the distance of the incipient C-C bond in the transition state, given in Å. Freq. refers to the imaginary frequency of the transition state. Energies are given in kcal/mol relative to the lowest energy substrate conformer, 'RR240'. Optimisation was conducted in Gaussian16 using the B3LYP 6-31G(d,p) functional and an implicit water solvent model.

Geom.	Name	Dihedral (°)	Distance (Å)	Freq. (Hz)	E_{rel} (reac)	E_{rel} (ts)	E_{rel} (prod)
Linear	RR	-100.1	2.10	-377.7	1.9	18.8	10.2
Linear	RR120	-86.8	2.11	-378.9	1.3	16.8	8.4
Linear	RR240	-86.7	2.06	-391.9	0	17.1	9.0
Linear	RS	-82.7	2.10	-384.3	4.6	17.3	9.1
Linear	RS120	-92.9	2.08	-388.8	2.4	16.7	7.9
Linear	RS240	-91.0	2.07	-407.7	1.2	18.4	10.8
Linear	SR	82.7	2.10	-384.3	3	17.3	9.1
Linear	SR120	91.0	2.07	-407.7	2.9	18.4	10.8
Linear	SR240	92.9	2.08	-388.8	2.1	16.7	7.9
Linear	SS	100.1	2.10	-377.7	1.9	18.8	10.2
Linear	SS120	86.3	2.06	-392.3	0.5	17.3	9.0
Linear	SS240	86.7	2.11	-376.8	0.9	16.4	8.4
Bent	RR	-94.3	2.14	-346.9	5.7	20.4	10.3
Bent	RR120	-81.7	2.16	-325.6	7.4	18.7	9.6
Bent	RR240	-89.9	2.12	-350.9	6.4	19.7	9.1
Bent	RS	-77.6	2.15	-337.9	6.6	19.0	9.5
Bent	RS120	-93.9	2.15	-336.6	6.0	19.4	9.7
Bent	RS240	-90.8	2.13	-365.3	6.8	20.2	11.4
Bent	SR	77.6	2.15	-337.9	6.7	19.0	9.5
Bent	SR120	90.8	2.13	-365.3	6.9	20.2	11.4
Bent	SR240	93.9	2.14	-336.6	6.1	19.4	9.9
Bent	SS	94.4	2.14	-349.4	5.8	20.2	10.7
Bent	SS120	89.9	2.12	-350.8	5.2	19.6	8.7
Bent	SS240	81.7	2.16	-345.6	7.6	18.7	9.6

Table S5. Number of frames meeting the NAC definition during MD simulations of 500ns (50,000 total frames per simulation). As in the main text, a NAC is defined as C-3 C-*beta* distances of less than or equal to 5 Å, with the previously described dihedral between -80° and -100° for pro-*(R)* NACs and between 80° and 100° for pro-*(S)* NACs.

LmrR_pAF_RMH			LmrR_pAF			LmrR_pAF_RGN		
**Replicate**	**Pro-*(R)***	**Pro-*(S)***	**Replicate**	**Pro-*(R)***	**Pro-*(S)***	**Replicate**	**Pro-*(R)***	**Pro-*(S)***
1	-	-	1	177	133	1	168	2186
2	1	-	2	3	1	2	46	53
3	4	35	3	550	25	3	448	1286
4	-	-	4	35	18	4	613	457
5	-	-	5	13	32	5	641	1671
6	-	-	6	-	-	6	584	1332
**Total**	5	35	**Total**	778	209	**Total**	2500	6985

Table S6. H-bond analysis performed with the cpptraj[4] analysis package, for **H-bond donors** interacting with the hemiaminal hydroxyl-moiety. Only the top three residues, with interactions of more than 100 total frames, are given. The intensity of green colour of the cells indicates the occurrence of the interaction. Cells highlighted yellow indicate residues introduced by directed evolution.

Variant	Hemiaminal	Replicate	Res	#	Res	#	Res	#
LmrR_pAF	*(R)*	1	W96	1027	-	-	-	-
LmrR_pAF	*(R)*	2	S95	146	W96	121	-	-
LmrR_pAF	*(R)*	3	N19	878	K22	537	-	-
LmrR_pAF	*(R)*	4	S95	1247	Q12	1108	W96	507
LmrR_pAF	*(R)*	5	N19	944	W96	756	-	-
LmrR_pAF	*(R)*	6	N19	2459	W96	280	K22	467
LmrR_pAF	*(S)*	1	Q12	7990	W96	812	S95	758
LmrR_pAF	*(S)*	2	W96	2631	N88	721	-	-
LmrR_pAF	*(S)*	3	W96	410	-	-	-	-
LmrR_pAF	*(S)*	4	W96	584	N88	470	-	-
LmrR_pAF	*(S)*	5	-	-	-	-	-	-
LmrR_pAF	*(S)*	6	-	-	-	-	-	-
RGN	*(R)*	1	R18	14329	N19	1962	-	-
RGN	*(R)*	2	N19	5023	R18	1684	-	-
RGN	*(R)*	3	W96	220	-	-	-	-
RGN	*(R)*	4	N19	716	N89	151	-	-
RGN	*(R)*	5	N89	4234	-	-	-	-
RGN	*(R)*	6	N89	3274	-	-	-	-
RGN	*(S)*	1	Q12	6508	W96	106	-	-
RGN	*(S)*	2	-	-	-	-	-	-
RGN	*(S)*	3	-	-	-	-	-	-
RGN	*(S)*	4	-	-	-	-	-	-
RGN	*(S)*	5	-	-	-	-	-	-
RGN	*(S)*	6	-	-	-	-	-	-
RMH-HIE	*(R)*	1	R92	2330	-	-	-	-
RMH-HIE	*(R)*	2	R92	720	-	-	-	-
RMH-HIE	*(R)*	3	H93	321	-	-	-	-
RMH-HIE	*(R)*	4	R92	310	-	-	-	-
RMH-HIE	*(R)*	5	R92	142	-	-	-	-
RMH-HIE	*(R)*	6	R92	15604	-	-	-	-

RMH-HIE	*(S)*	1	R92	5893	-	-	-	-
RMH-HIE	*(S)*	2	R92	1850	-	-	-	-
RMH-HIE	*(S)*	3	R92	1009	-	-	-	-
RMH-HIE	*(S)*	4	W96	2993	Q12	1149	S95	906
RMH-HIE	*(S)*	5	H93	167	-	-	-	-
RMH-HIE	*(S)*	6	R92	2147	H93	224	-	-
RMH-HID	*(R)*	1	H93	3752	-	-	-	-
RMH-HID	*(R)*	2	H93	530	R92	178	-	-
RMH-HID	*(R)*	3	R92	386	H93	162	-	-
RMH-HID	*(R)*	4	H93	5923	R92	335	-	-
RMH-HID	*(R)*	5	H93	3249	-	-	-	-
RMH-HID	*(R)*	6	R92	779	H93	207	-	-
RMH-HID	*(S)*	1	R92	8926	H93	1348	-	-
RMH-HID	*(S)*	2	H93	841	-	-	-	-
RMH-HID	*(S)*	3	-	-	-	-	-	-
RMH-HID	*(S)*	4	H93	1579	R92	285	-	-
RMH-HID	*(S)*	5	H93	3090	R92	347	-	-
RMH-HID	*(S)*	6	H93	1978	S97	252	-	-
RMH-HIP	*(R)*	1	H93	556	R92	553	-	-
RMH-HIP	*(R)*	2	H93	899	-	-	-	-
RMH-HIP	*(R)*	3	H93	340	-	-	-	-
RMH-HIP	*(R)*	4	R92	1028	H93	213	-	-
RMH-HIP	*(R)*	5	R92	1369	H93	297	-	-
RMH-HIP	*(R)*	6	R92	1152	H93	390	-	-
RMH-HIP	*(S)*	1	R92	1128	H93	231	-	-
RMH-HIP	*(S)*	2	R92	5655	H93	621	-	-
RMH-HIP	*(S)*	3	R92	3565	H93	301	-	-
RMH-HIP	*(S)*	4	H93	1174	-	-	-	-
RMH-HIP	*(S)*	5	-	-	-	-	-	-
RMH-HIP	*(S)*	6	H93	594	R92	514	-	-

Table S7. H-bond analysis performed with the cpptraj analysis package, for **H-bond acceptors** interacting with the hemiaminal hydroxyl-moiety. Only the top three residues, with interactions of more than 100 total frames, are given. The intensity of green colour of the cells indicates the occurrence of the interaction. Cells highlighted yellow indicate residues introduced by directed evolution.

Variant	Hemiaminal	Replicate	Res	#	Res	#	Res	#
LmrR_pAF	*(R)*	1	F93	3515	S95	1923	-	-
LmrR_pAF	*(R)*	2	S95	3640	L91	766	-	-
LmrR_pAF	*(R)*	3	N19	2101	-	-	-	-
LmrR_pAF	*(R)*	4	Q12	32154	S95	782	-	-
LmrR_pAF	*(R)*	5	D100	992	N19	431	-	-
LmrR_pAF	*(R)*	6	N19	2920	-	-	-	-
LmrR_pAF	*(S)*	1	Q12	8732	S95	5287	-	-
LmrR_pAF	*(S)*	2	N88	954	E7	138	-	-
LmrR_pAF	*(S)*	3	D100	37196	-	-	-	-
LmrR_pAF	*(S)*	4	N88	13242	L91	1346	N88	309
LmrR_pAF	*(S)*	5	D100	24605	S97	164	-	-
LmrR_pAF	*(S)*	6	D100	38997	-	-	-	-
RGN	*(R)*	1	N19	23941	I16	165	-	-
RGN	*(R)*	2	A92	644	N89	111	-	-
RGN	*(R)*	3	D100	6642	-	-	-	-
RGN	*(R)*	4	N89	1402	D100	139	-	-
RGN	*(R)*	5	D100	3099	A92	1005	N89	241
RGN	*(R)*	6	N89	122	-	-	-	-
RGN	*(S)*	1	D100	4912	Q12	292	-	-
RGN	*(S)*	2	D100	3002	N89	310	-	-
RGN	*(S)*	3	D100	29765	-	-	-	-
RGN	*(S)*	4	D100	43735	-	-	-	-
RGN	*(S)*	5	D100	40410	-	-	-	-
RGN	*(S)*	6	D100	42434	-	-	-	-
RMH-HIE	*(R)*	1	H93	1374	D100	842	-	-
RMH-HIE	*(R)*	2	H93	741	D100	330	-	-
RMH-HIE	*(R)*	3	H93	2126	D100	819	-	-
RMH-HIE	*(R)*	4	H93	6372	D100	754	-	-
RMH-HIE	*(R)*	5	H93	2329	D100	717	-	-
RMH-HIE	*(R)*	6	D100	361	-	-	-	-

RMH-HIE	*(S)*	1	D100	3378	H93	1398	M89	564
RMH-HIE	*(S)*	2	H93	2033	D100	1248	M89	610
RMH-HIE	*(S)*	3	D100	7312	H93	3684	M89	1587
RMH-HIE	*(S)*	4	M19	1981	N88	372	S95	328
RMH-HIE	*(S)*	5	D100	9396	H93	652	M89	169
RMH-HIE	*(S)*	6	D100	14080	H93	810	M89	189
RMH-HID	*(R)*	1	H93	827	D100	141	-	-
RMH-HID	*(R)*	2	D100	1562	H93	973	-	-
RMH-HID	*(R)*	3	H93	2809	D100	1407	-	-
RMH-HID	*(R)*	4	D100	630	S95	118	-	-
RMH-HID	*(R)*	5	H93	756	R92	208	-	-
RMH-HID	*(R)*	6	D100	7923	H93	1467	-	-
RMH-HID	*(S)*	1	M89	1713	D100	1929	H93	359
RMH-HID	*(S)*	2	D100	35877	-	-	-	-
RMH-HID	*(S)*	3	D100	9332	H93	1892	-	-
RMH-HID	*(S)*	4	D100	2020	H93	539	-	-
RMH-HID	*(S)*	5	D100	7832	H93	763	M89	320
RMH-HID	*(S)*	6	D100	22672	H93	231	-	-
RMH-HIP	*(R)*	1	D100	356	-	-	-	-
RMH-HIP	*(R)*	2	M89	414	R92	244	-	-
RMH-HIP	*(R)*	3	R92	344	D100	168	-	-
RMH-HIP	*(R)*	4	D100	618	R93	149	M89	117
RMH-HIP	*(R)*	5	D100	102	-	-	-	-
RMH-HIP	*(R)*	6	D100	653	-	-	-	-
RMH-HIP	*(S)*	1	D100	3862	M89	291	-	-
RMH-HIP	*(S)*	2	D100	1619	M89	299	-	-
RMH-HIP	*(S)*	3	D100	1476	-	-	-	-
RMH-HIP	*(S)*	4	D100	170	-	-	-	-
RMH-HIP	*(S)*	5	D100	17587	-	-	-	-
RMH-HIP	*(S)*	6	D100	2342	M89	116	-	-

Supporting Figures

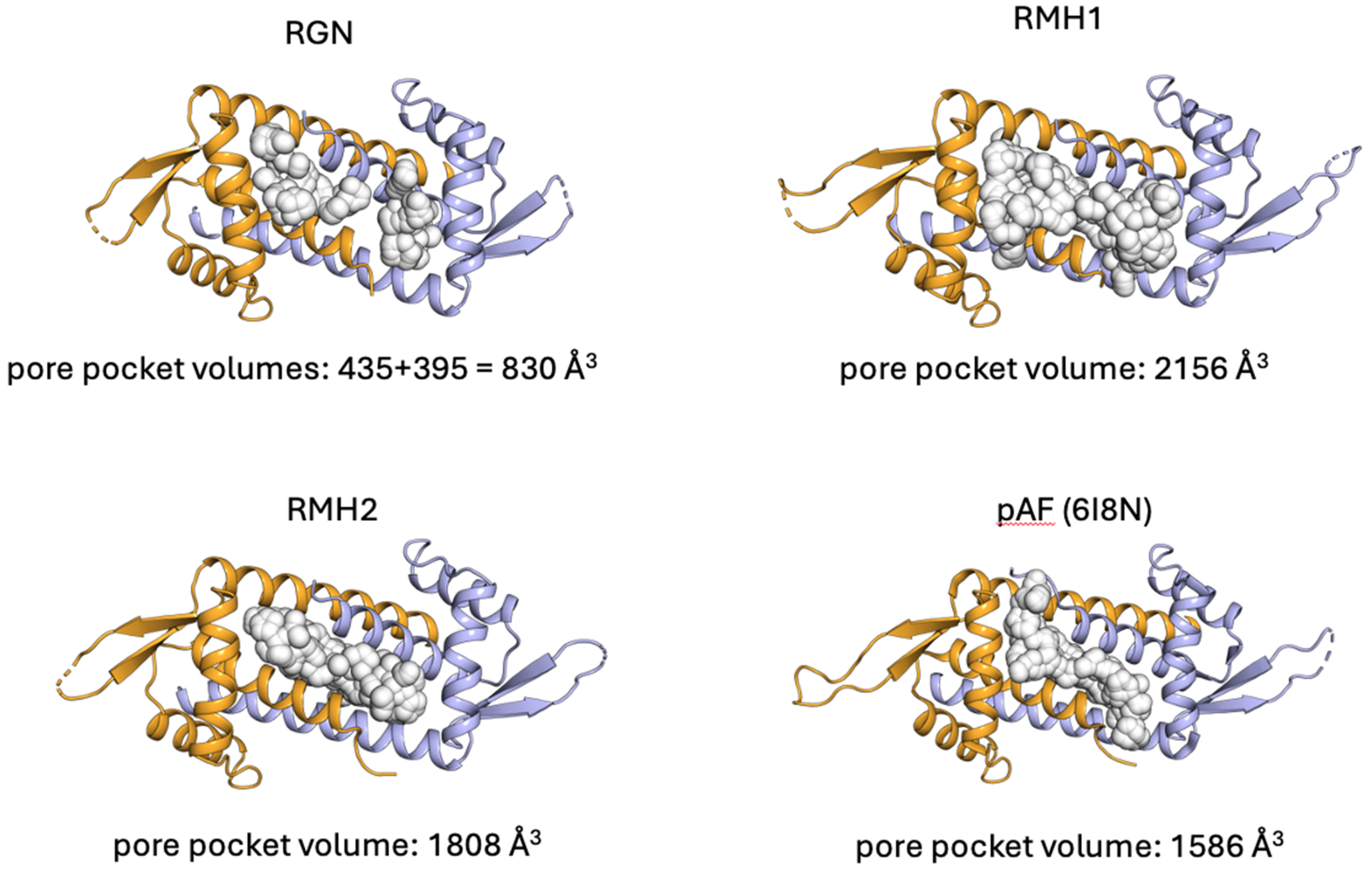

Figure S1: Pocket volumes for the crystal structures analysed herein, calculated with PyVOL[5] version 1.7.6. Default settings: minimum probe radius = 1.4 Å, maximum probe radius = 3.4 Å.

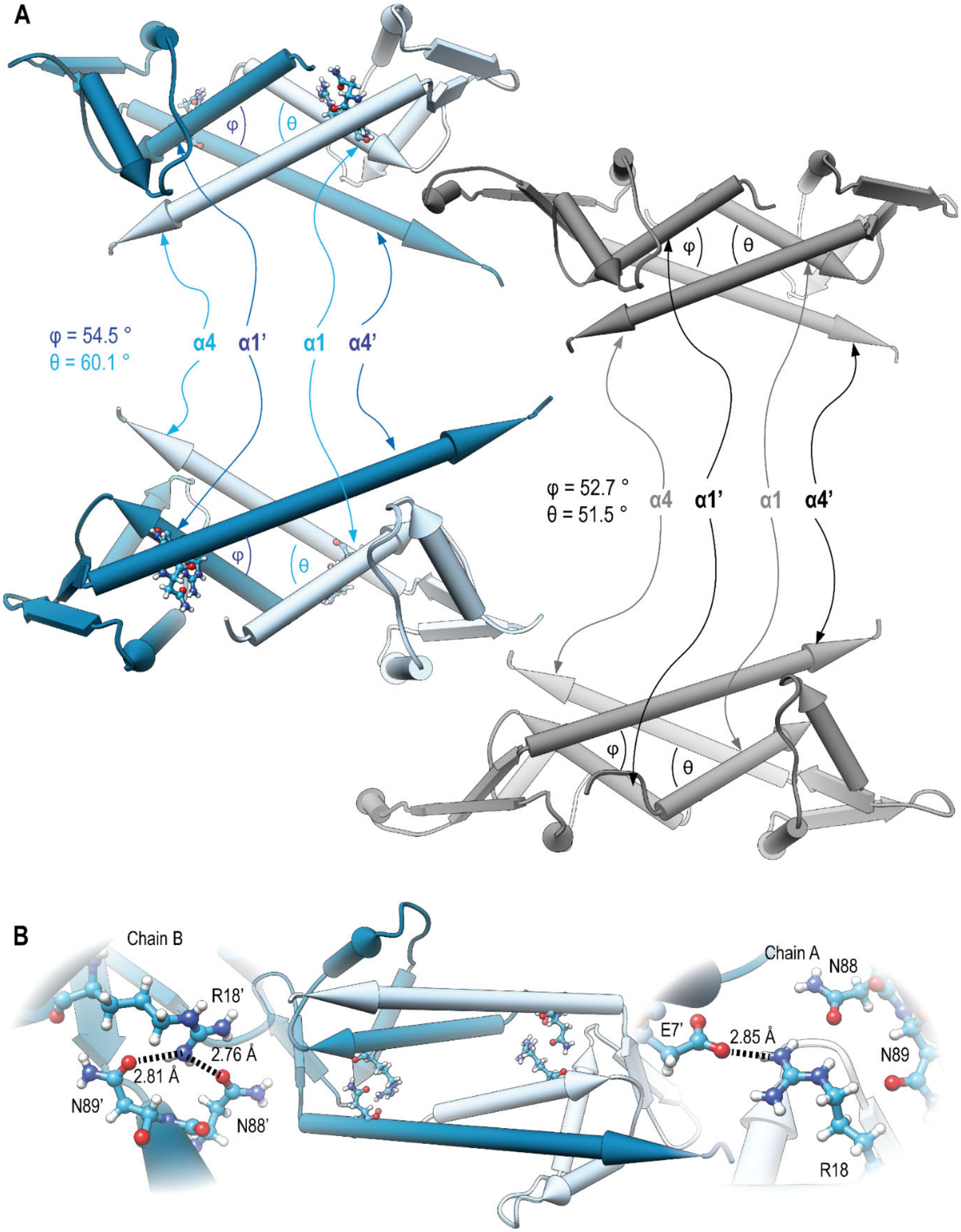

Figure S2: A. Inter-helix angles between the α1 and α4 helices in LmrR_pAF (PDB: 6I8N; grey) and LmrR_pAF_RGN (PDB: 9GKT; blue) showing the key interactions of the residues introduced during directed evolution. **B**. Close-up view of the H-bonding interactions with the R18 sidechain in each monomer of LmrR_pAF_RGN.

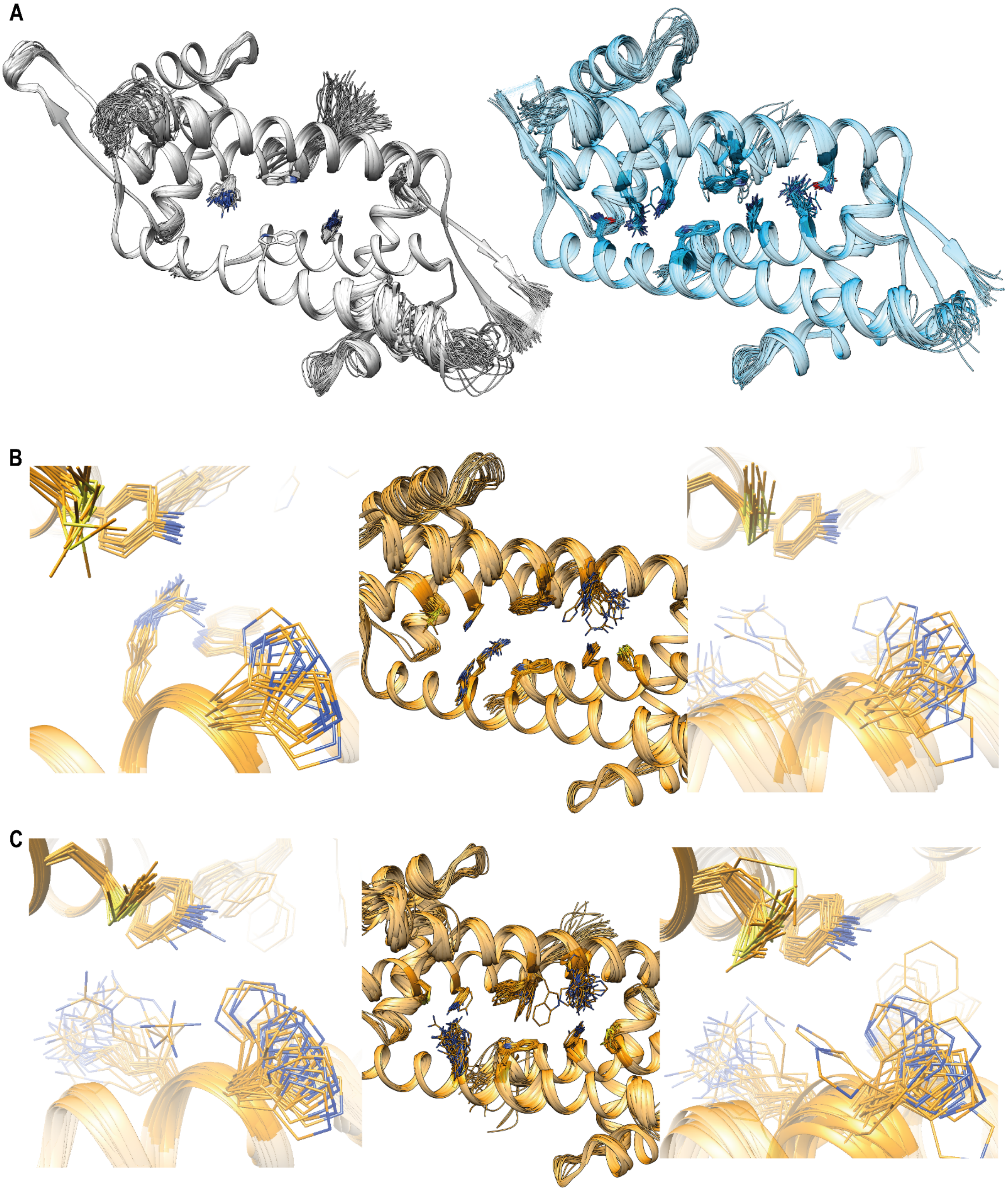

Figure S3: A.Ensemble refinement structures obtained with LmrR_pAF (PDB: 6I8N; grey) and LmrR_pAF_RGN (PDB: 9GKT; blue). **B**. Ensemble refinement structures obtained with LmrR_pAF_RMH crystal 1 with close-up of the active site in each monomer (PDB: 9GKR). **C**. Ensemble refinement structures obtained with LmrR_pAF_RMH crystal 2 with close-up of the active site in each monomer (PDB: 9GKS).

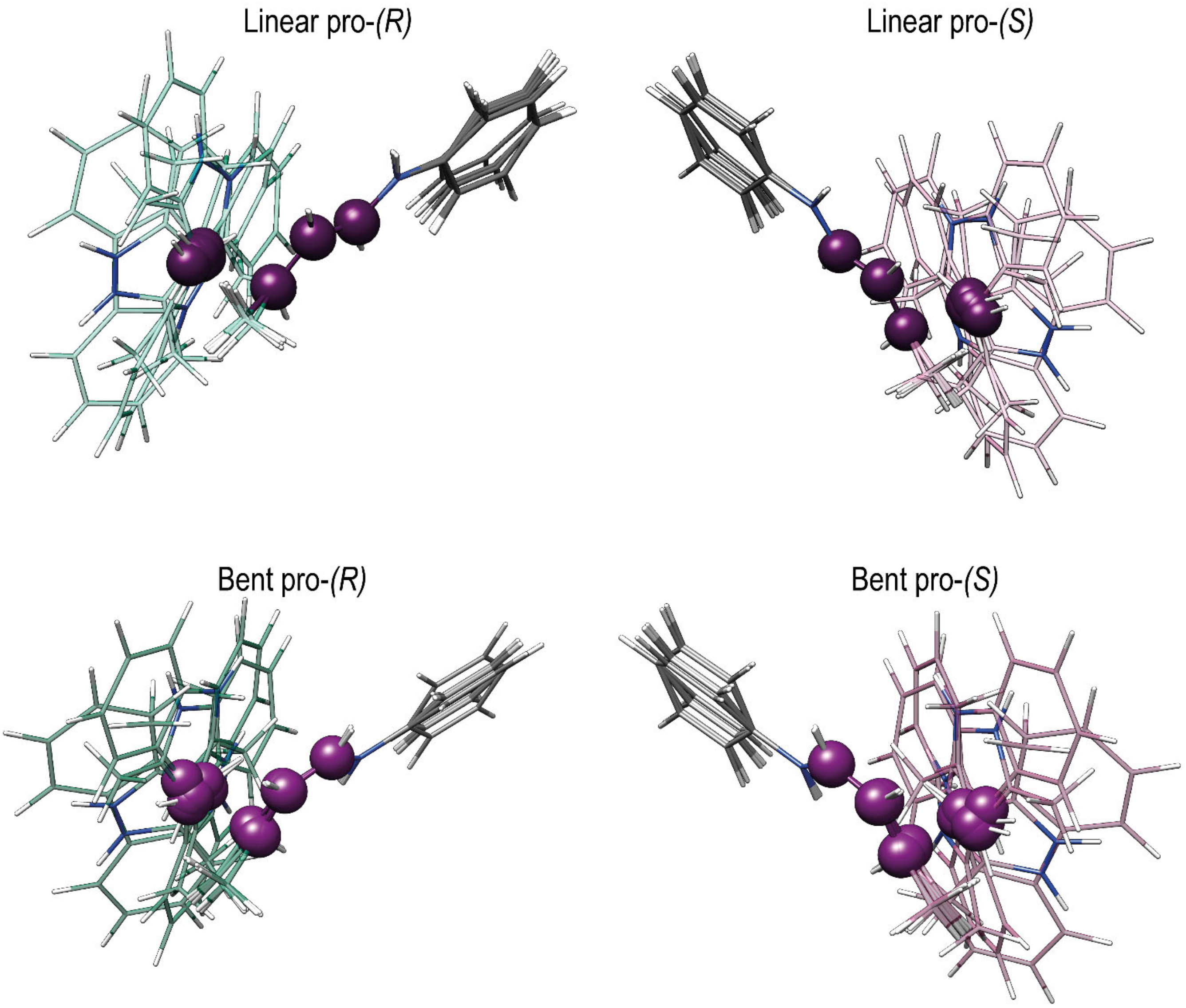

Figure S4: Overlay of the transition state structures calculation for the truncated model of the Friedel-Crafts reaction. pAF is simplified to aniline, and hexenal to crotonaldehyde. In this way, spurious energy differences between transition states due to different conformations of the flexible moieties was avoided. We permutated the face of the iminium ion that the 2-methyl-indole substrate approaches (pro-(R) or pro-(S)), the dihedral angle around the incipient C-C bond (3 staggered conformations), the face of the indole which attacks the β-carbon (pro-(R) or pro-(S) – a fleeting chiral centre that is abolished after re-aromatisation) as well as the relative conformation of the two double-bonds in the iminium ion (linear or bent). This produced a total of 24 transition states, 12 pro-*(R)* (green) and 12 pro-*(S)* (pink).

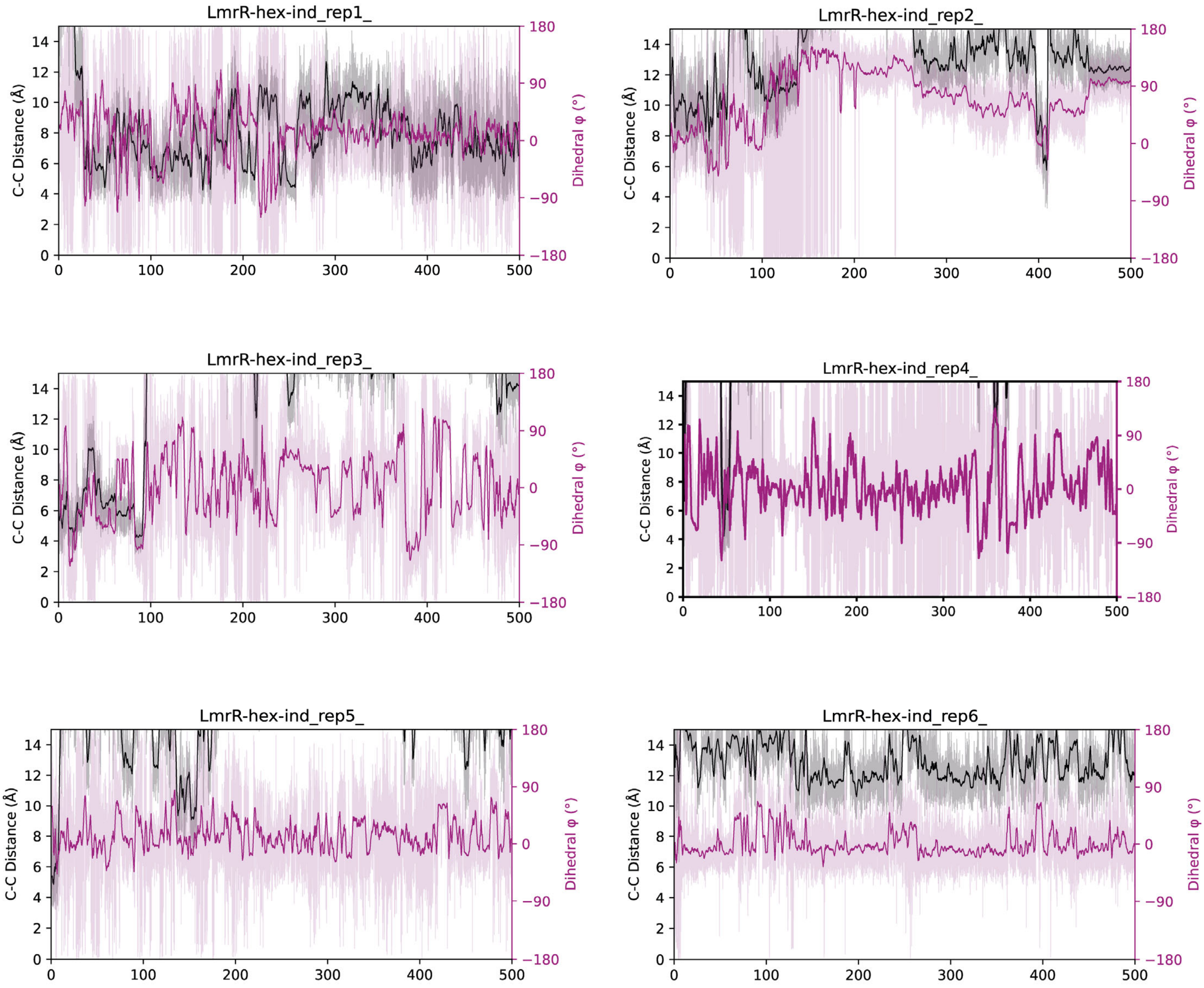

Figure S5: C-C distances (black) and dihedral angles (purple, as described in the main text) for the MD NAC simulations of the Friedel-Crafts reaction with the LmrR_pAF (parent) variant over 6 replicate simulations with 500 ns each. Distances over 15 Å are not relevant for catalysis and therefore not shown.

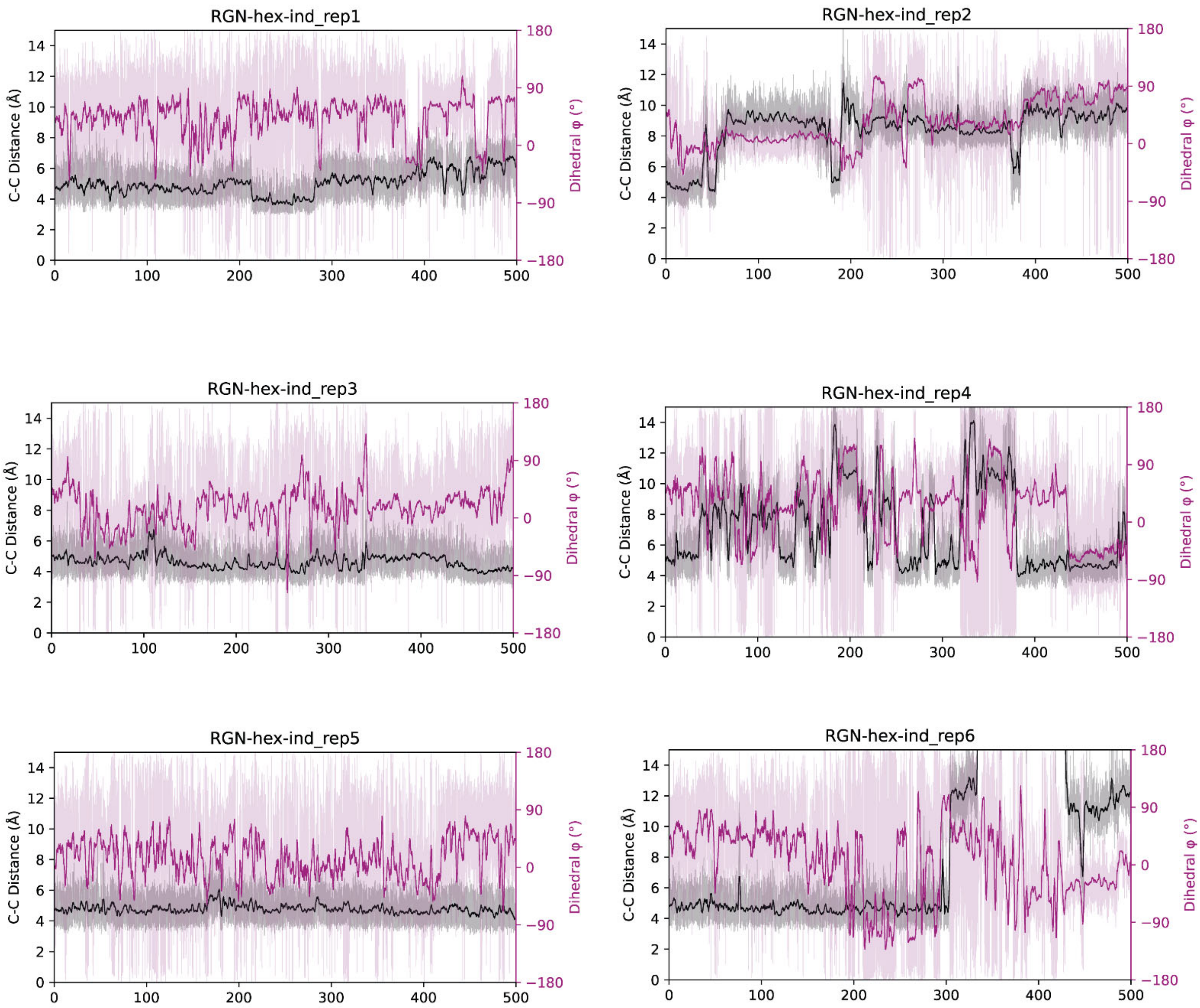

Figure S6: C-C distances (black) and dihedral angles (purple, as described in the main text) for the MD NAC simulations of the Friedel-Crafts reaction with the LmrR_pAF_RGN variant over 6 replicate simulations with 500 ns each. Distances over 15 Å are not relevant for catalysis and therefore not shown.

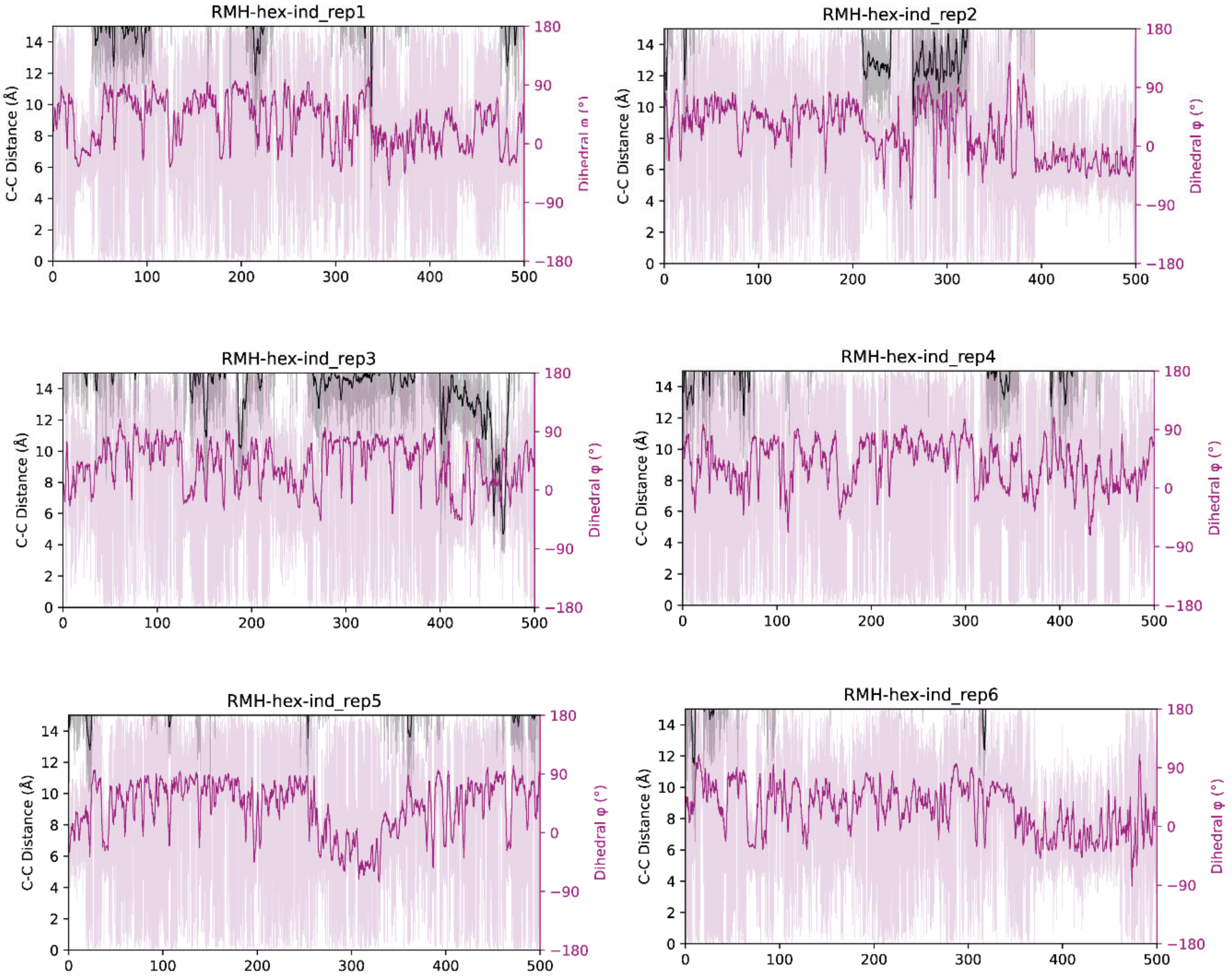

Figure S7: C-C distances (black) and dihedral angles (purple, as described in the main text) for the MD NAC simulations of the Friedel-Crafts reaction with the LmrR_pAF_RMH variant over 6 replicate simulations with 500 ns each. Distances over 15 Å are not relevant for catalysis and therefore not shown.

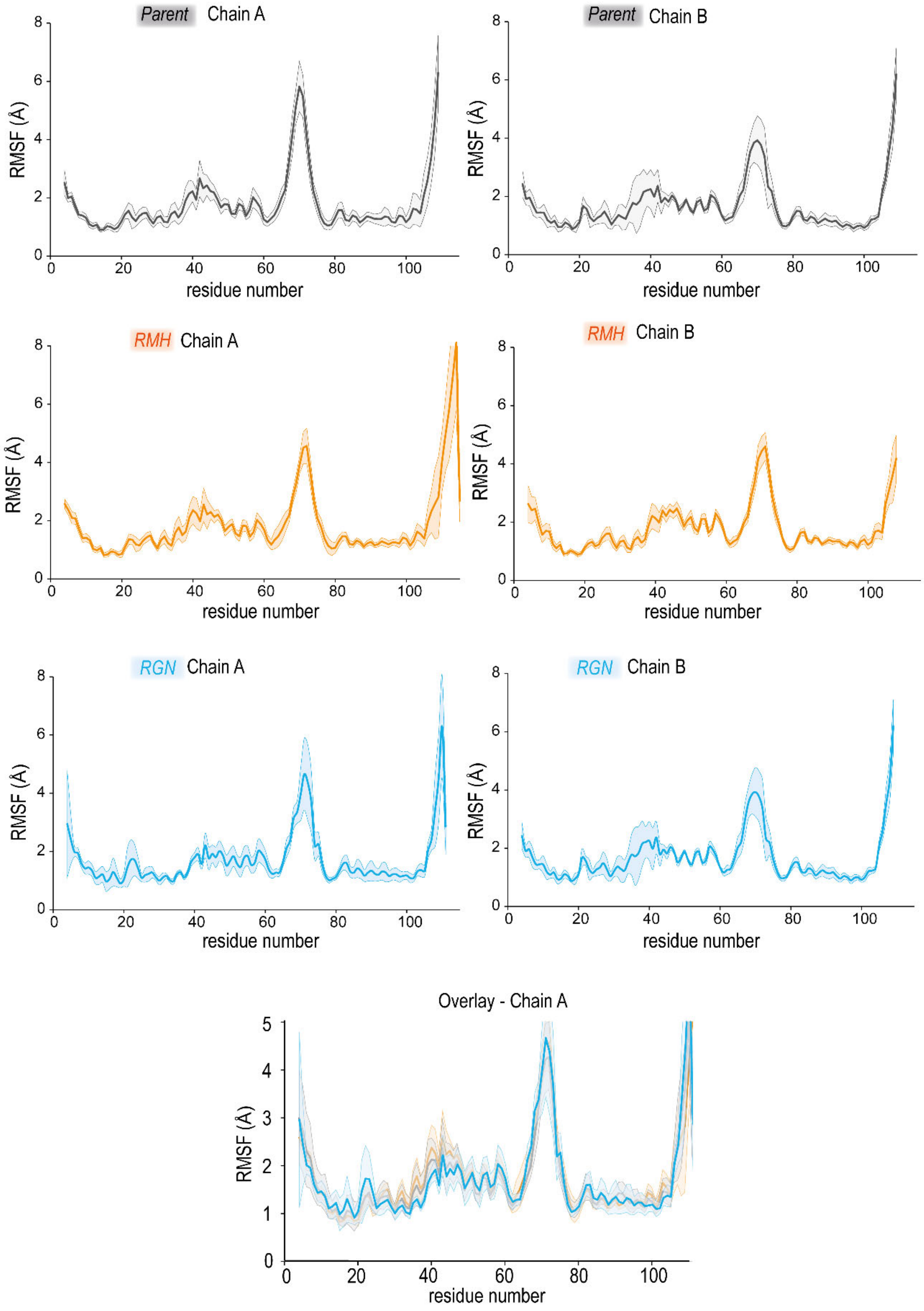

Figure S8: RMSF calculations performed with the mdtraj[6] package on the Friedel-Crafts MD NAC simulations for all Cα atoms. The solid line represents the average over 6 replicates whilst the lighter coloured region shows the standard deviation.

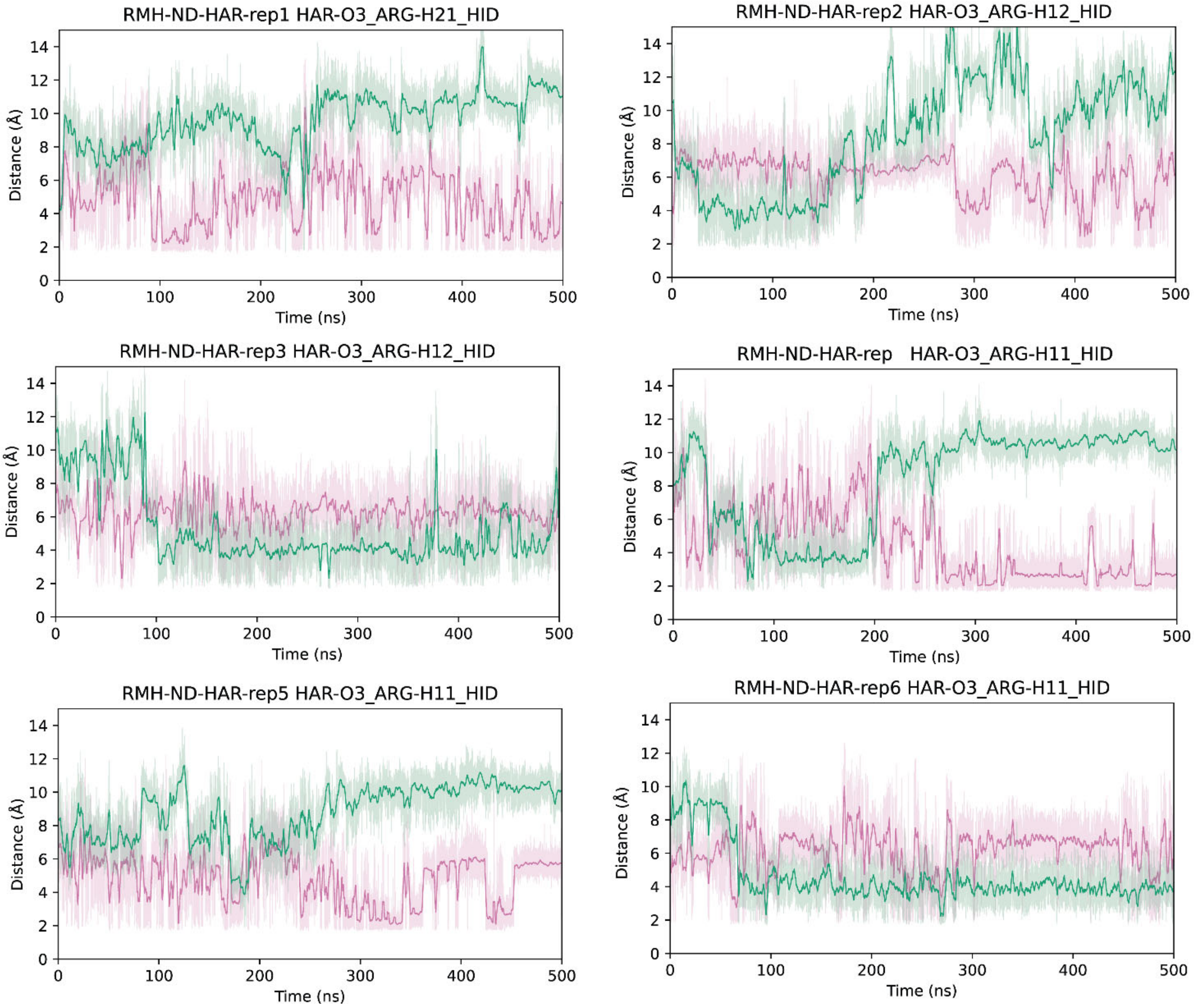

Figure S9: Measurements of the distances between hemiaminal-O-R92(H-bond donor atom) (green pale line, solid line is 2ns average) and hemiaminal-O-H93(δH) (pink pale line, solid line is 2ns average) atoms along 6 replicates 500ns simulation. In these simulations the hemiaminal had the *(R)* configuration and H93 was in the δ-protonation state.

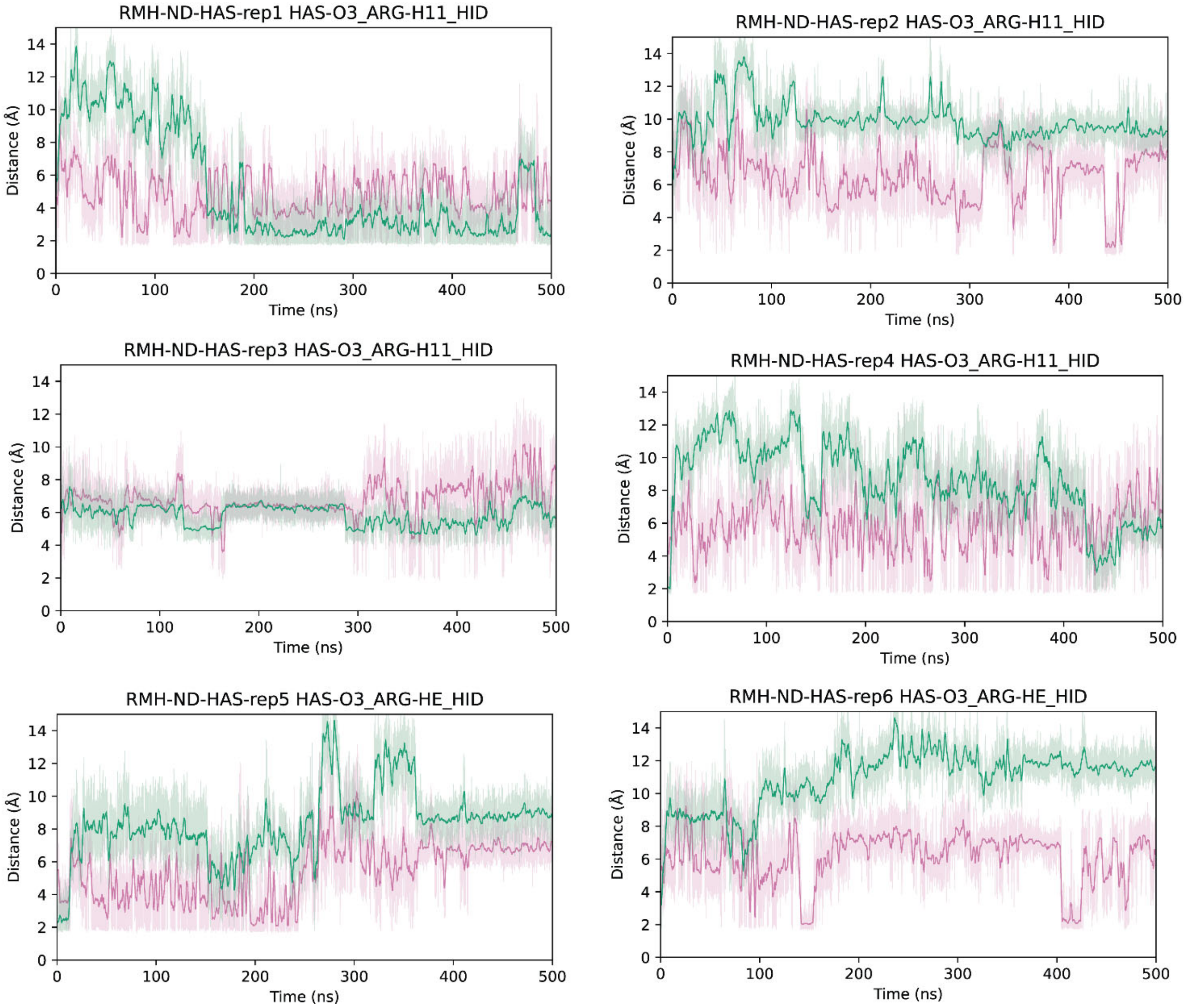

Figure S10: Measurements of the distances between hemiaminal-O-R92(H-bond donor atom) (green pale line, solid line is 2ns average) and hemiaminal-O-H93(δH) (pink pale line, solid line is 2ns average) atoms along 6 replicates 500ns simulation. In these simulations the hemiaminal had the *(S)* configuration and H93 was in the δ-protonation state.

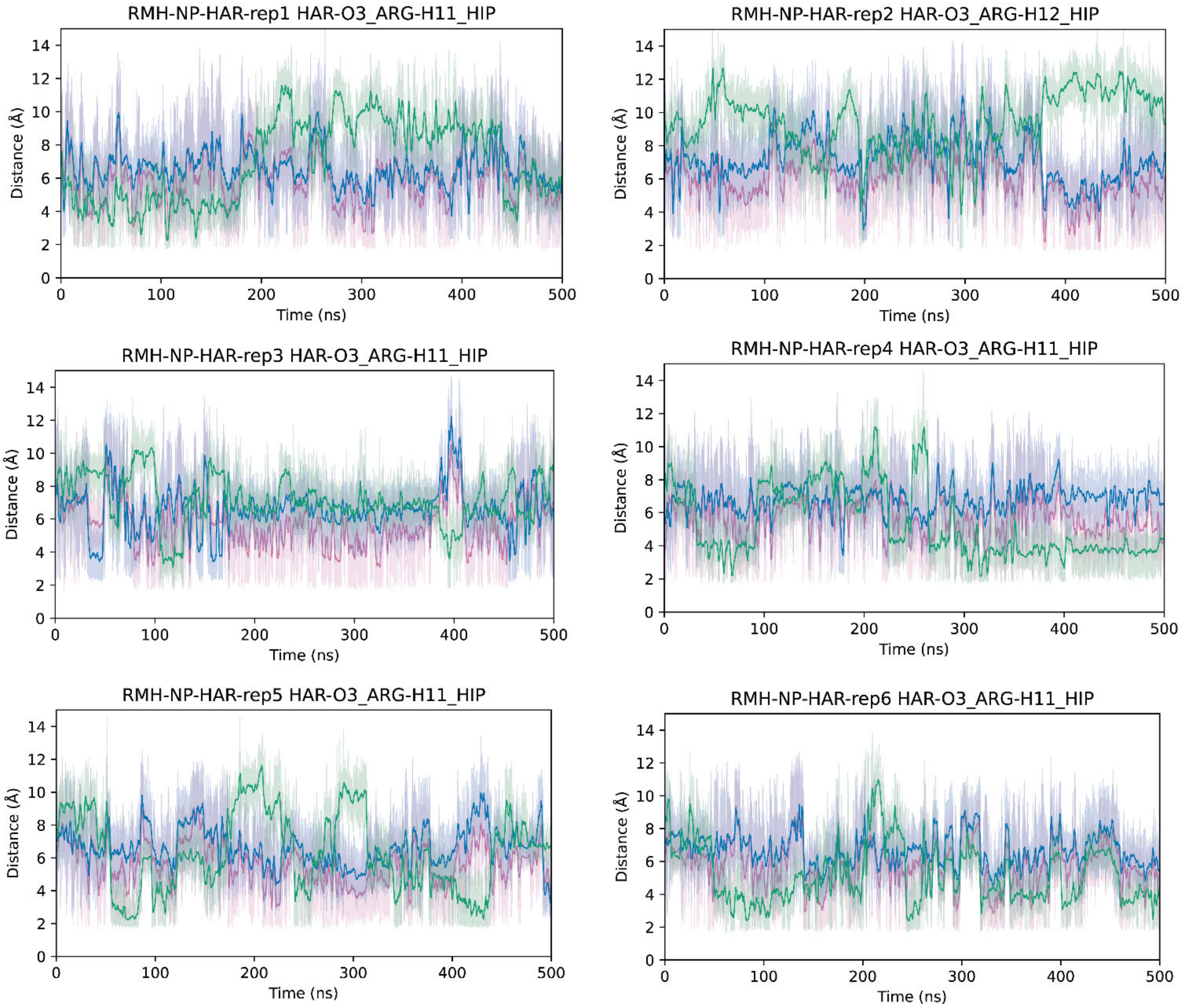

Figure S11: Measurements of the distances between hemiaminal-O-R92(H-bond donor atom) (green pale line, solid line is 2ns average) and hemiaminal-O-H93(δH) (pink pale line, solid line is 2ns average) and hemiaminal-O-H93(εH) (blue pale line, solid line is 2ns average) atoms along 6 replicates 500ns simulation. In these simulations the hemiaminal had the *(R)* configuration and H93 was in the double-protonation state.

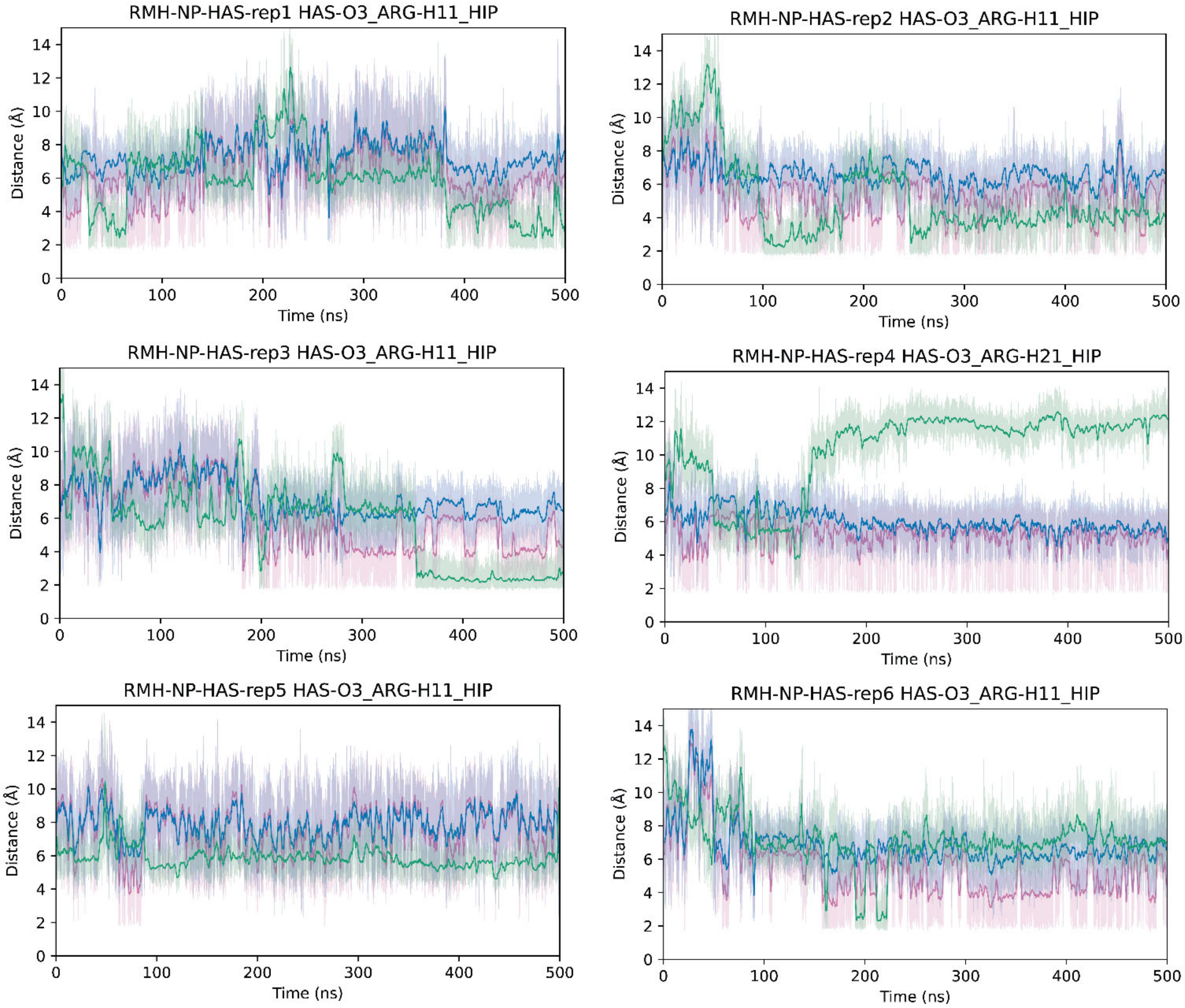

Figure S12: Measurements of the distances between hemiaminal-O-R92(H-bond donor atom) (green pale line, solid line is 2ns average) and hemiaminal-O-H93(δH) (pink pale line, solid line is 2ns average) and hemiaminal-O-H93(εH) (blue pale line, solid line is 2ns average) atoms along 6 replicates 500ns simulation. In these simulations the hemiaminal had the *(S)* configuration and H93 was in the double-protonation state.

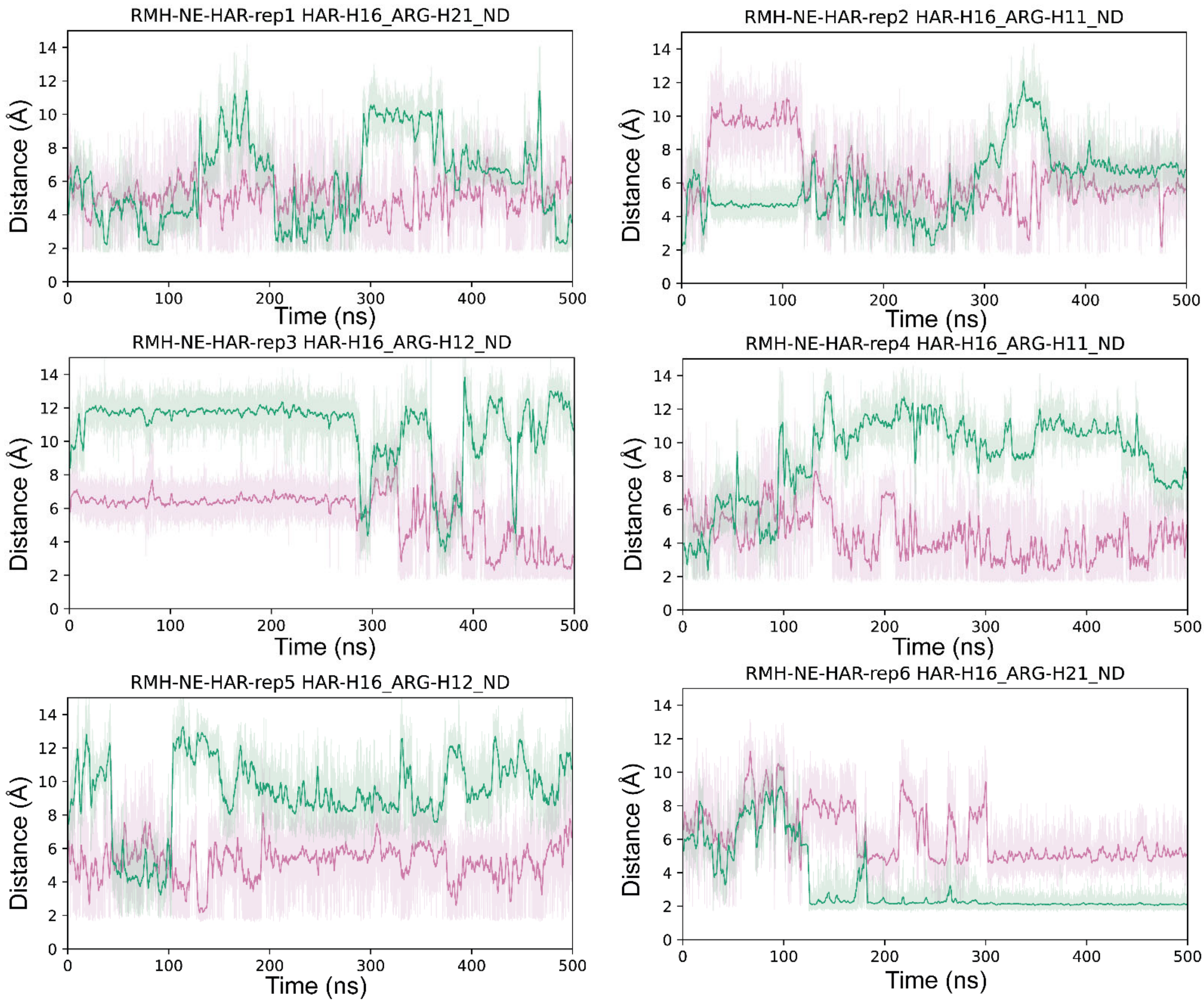

Figure S13: Measurements of the distances between hemiaminal-O-R92(H-bond donor atom) (green pale line, solid line is 2ns average) and hemiaminal-OH-H93(δN) (pink pale line, solid line is 2ns average) along 6 replicates 500ns simulation. In these simulations the hemiaminal had the *(R)* configuration and H93 was in the ε-protonation state.

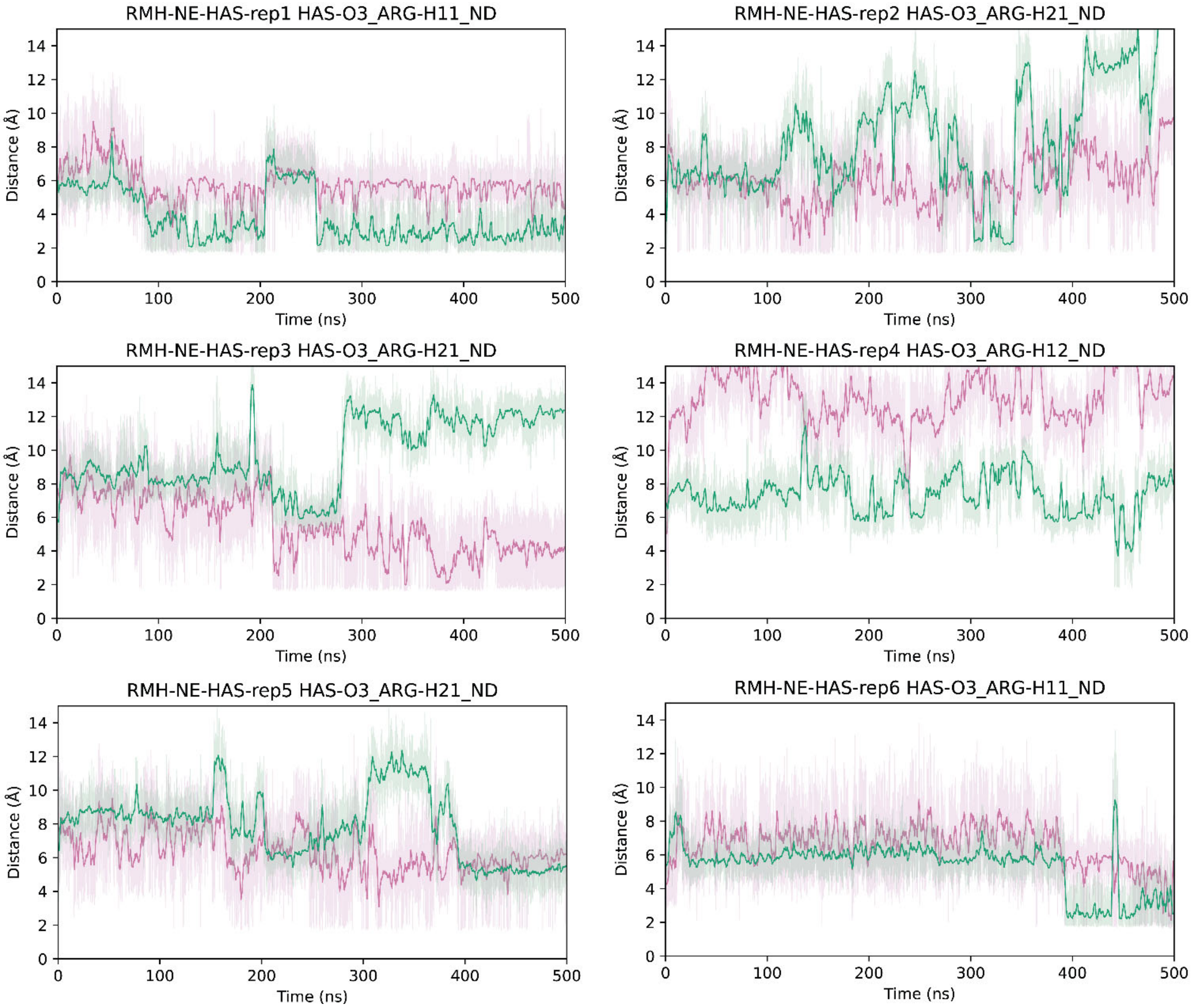

Figure S14: Measurements of the distances between hemiaminal-O-R92(H-bond donor atom) (green pale line, solid line is 2ns average) and hemiaminal-OH-H93(δN) (pink pale line, solid line is 2ns average) along 6 replicates 500ns simulation. In these simulations the hemiaminal had the *(R)* configuration and H93 was in the ε-protonation state.

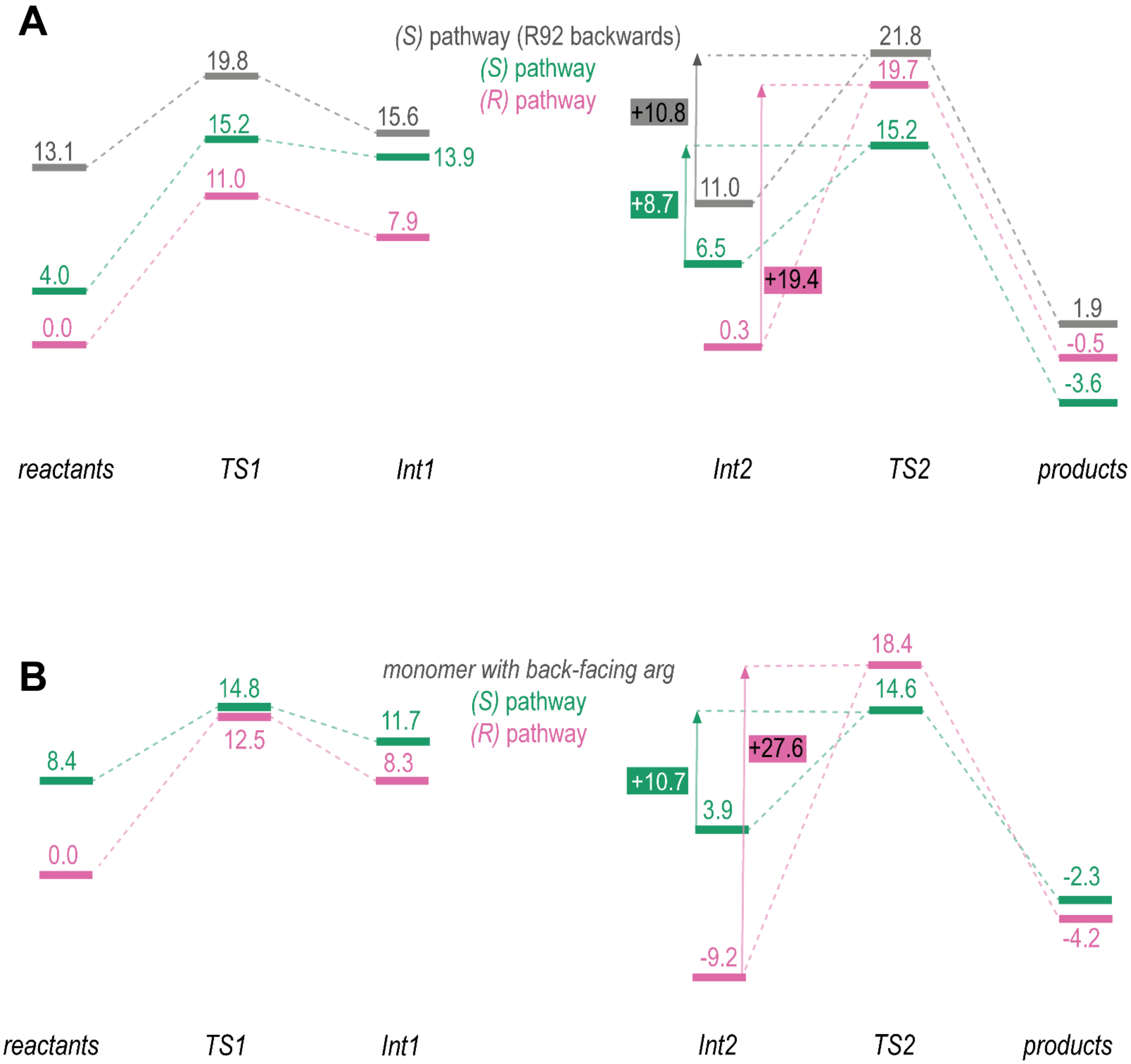

Figure S15: Energies of the quantum cluster models of the LmrR_pAF_RMH variant produced from residues pAF15, M19, M89, R92, H93, W96, V99, D100 and I103 in the monomer with forward-facing R92 (**A**) and the monomer with backward-facing R92 (**B**). Structures were optimised with the Gaussian 16 software using the B3LYP 6-31G(d,p) functional and intermediates were verified to have no imaginary frequencies, and transition states to have exactly one imaginary frequency corresponding to a vibration along the reaction coordinate. The maximum and minimum of the imaginary vibrations in the transition states were then optimised to locate the reactant and product structures. Finally, the energies were recalculated with the B3LYP 6-311G++(2d,2p) functional, which produced the energies shown in the figure, in units of kcal/mol.

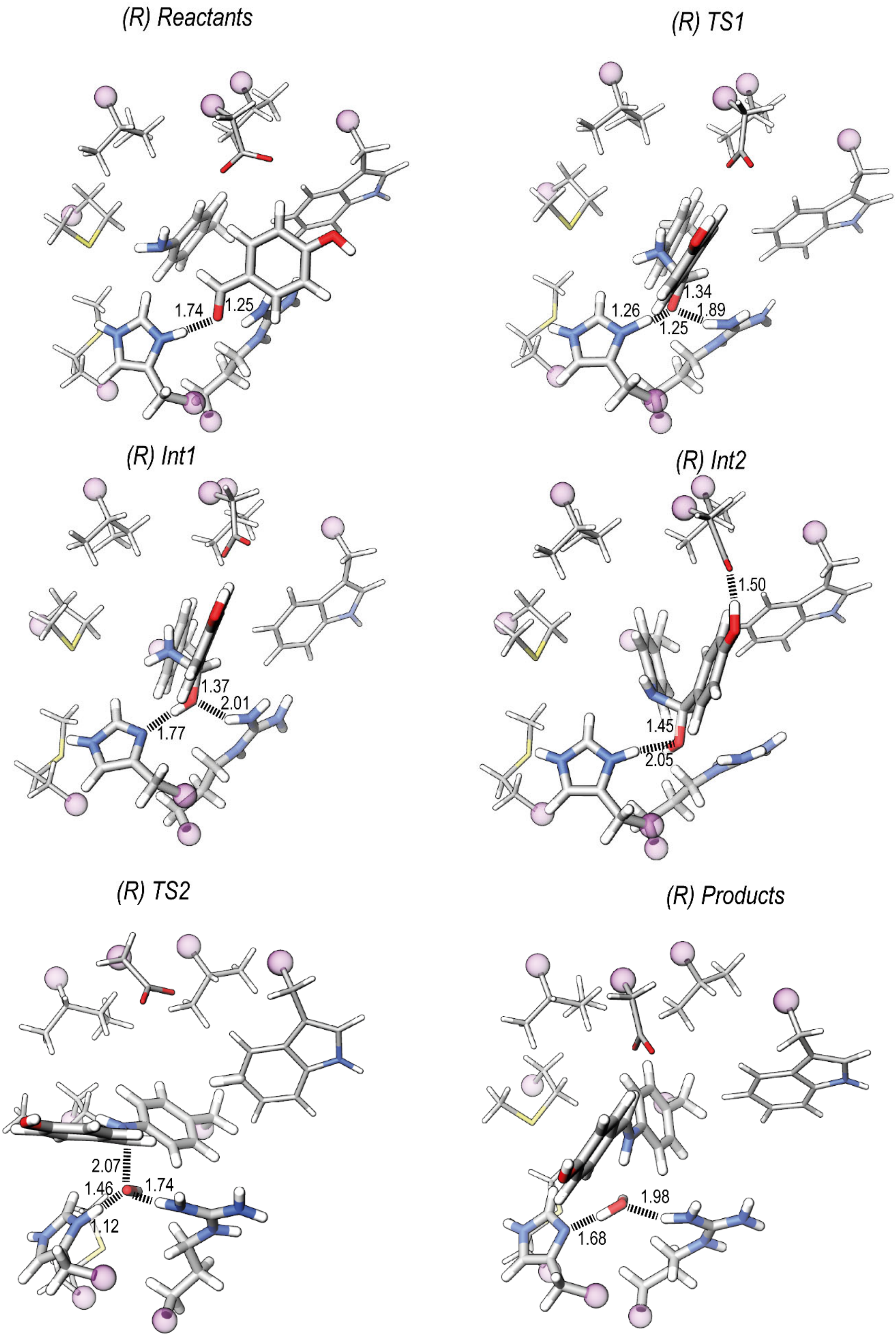

Figure S16: Structures of the intermediates and transition states for the quantum cluster model of the *(R)*-configured pathway produced from the LmrR_pAF_RMH monomer with forward facing R92 residue. Key bond lengths and interactions are labelled in Å units.

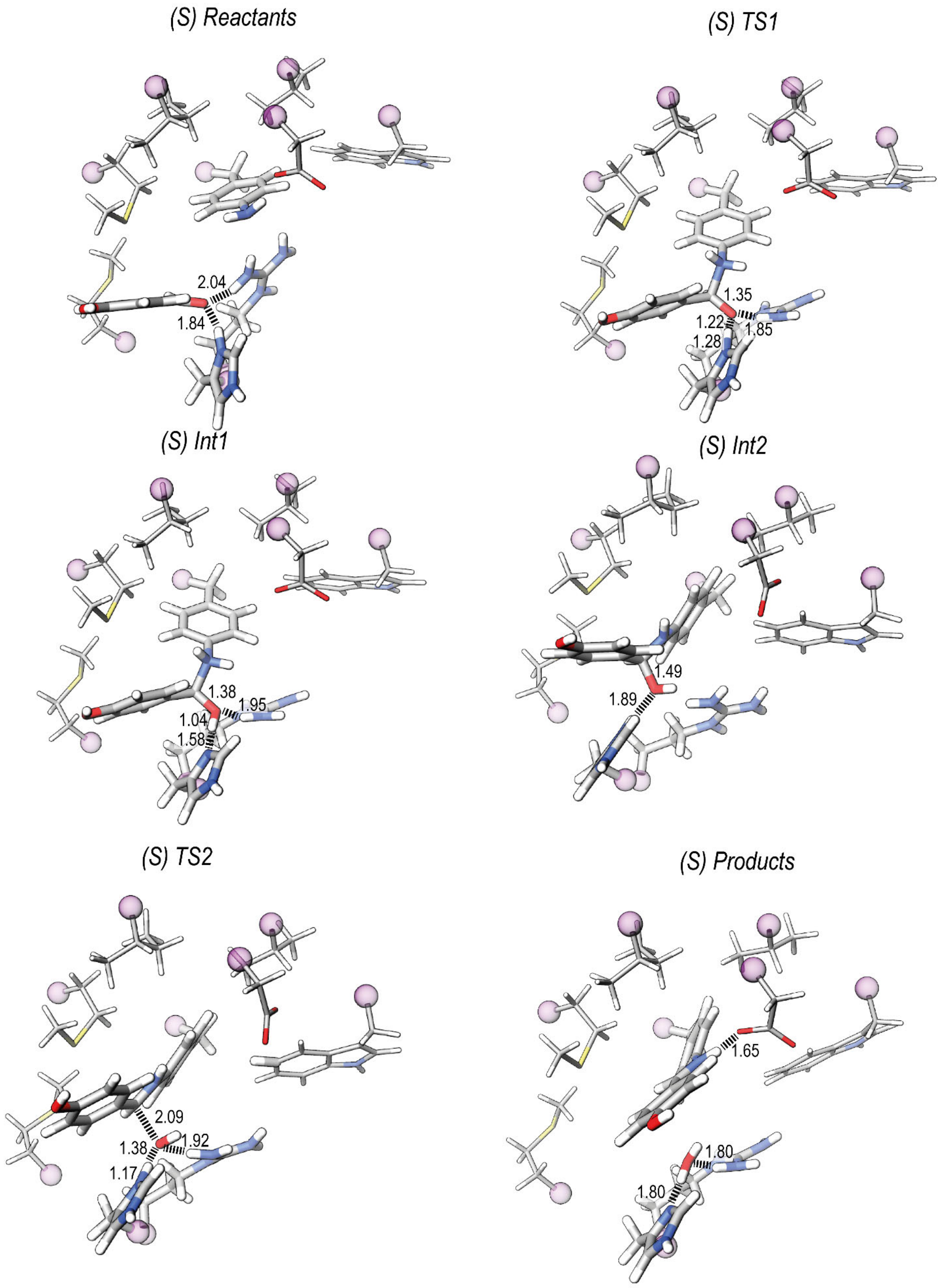

Figure S17: Structures of the intermediates and transition states for the quantum cluster model of the *(S)*-configured pathway produced from the LmrR_pAF_RMH monomer with forward facing R92 residue. Key bond lengths and interactions are labelled in Å units.

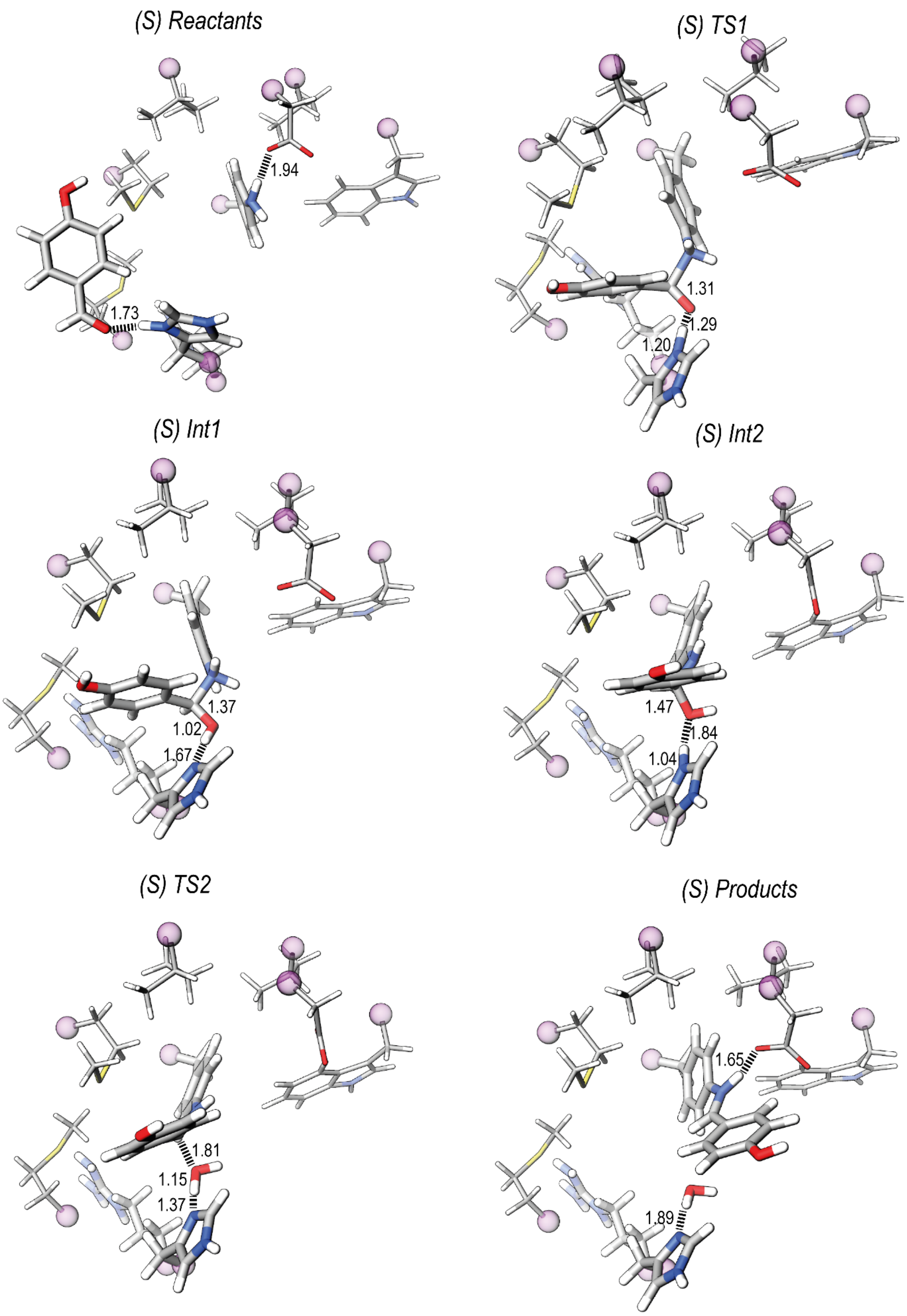

Figure S18: Structures of the intermediates and transition states for the quantum cluster model of the *(S)*-configured pathway produced from the LmrR_pAF_RMH monomer with forward facing R92 residue, however the conformation of this residue was adjusted to match the backward-facing conformation found in the other monomer. Key bond lengths and interactions are labelled in Å units.

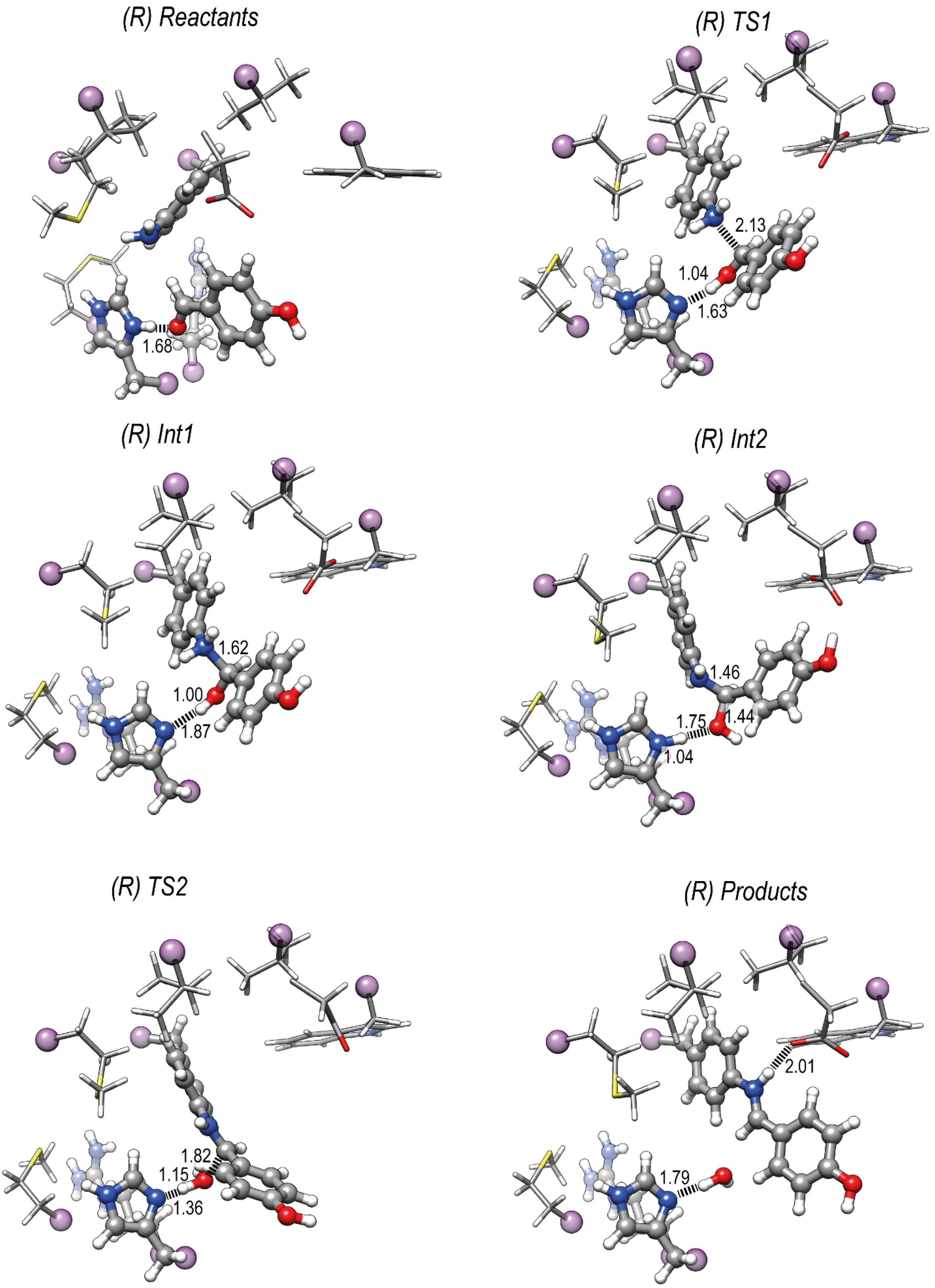

Figure S19: Structures of the intermediates and transition states for the quantum cluster model of the *(R)*-configured pathway produced from the LmrR_pAF_RMH monomer with backward facing R92 residue. Key bond lengths and interactions are labelled in Å units.

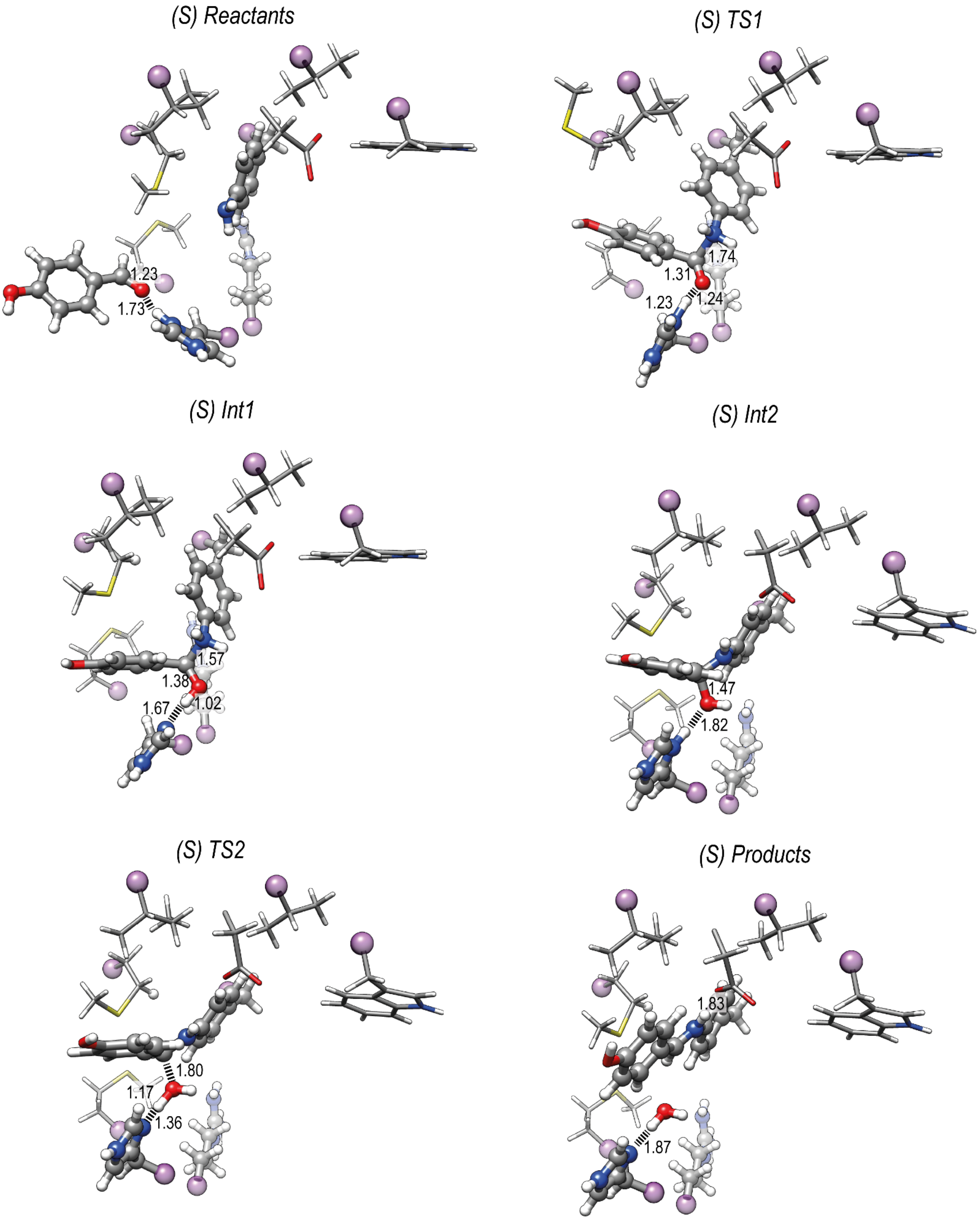

Figure S20: Structures of the intermediates and transition states for the quantum cluster model of the *(S)*-configured pathway produced from the LmrR_pAF_RMH monomer with backward facing R92 residue. Key bond lengths and interactions are labelled in Å units.

pAF_hemiaminal MD simulations cation-π interactions R92-pAF15

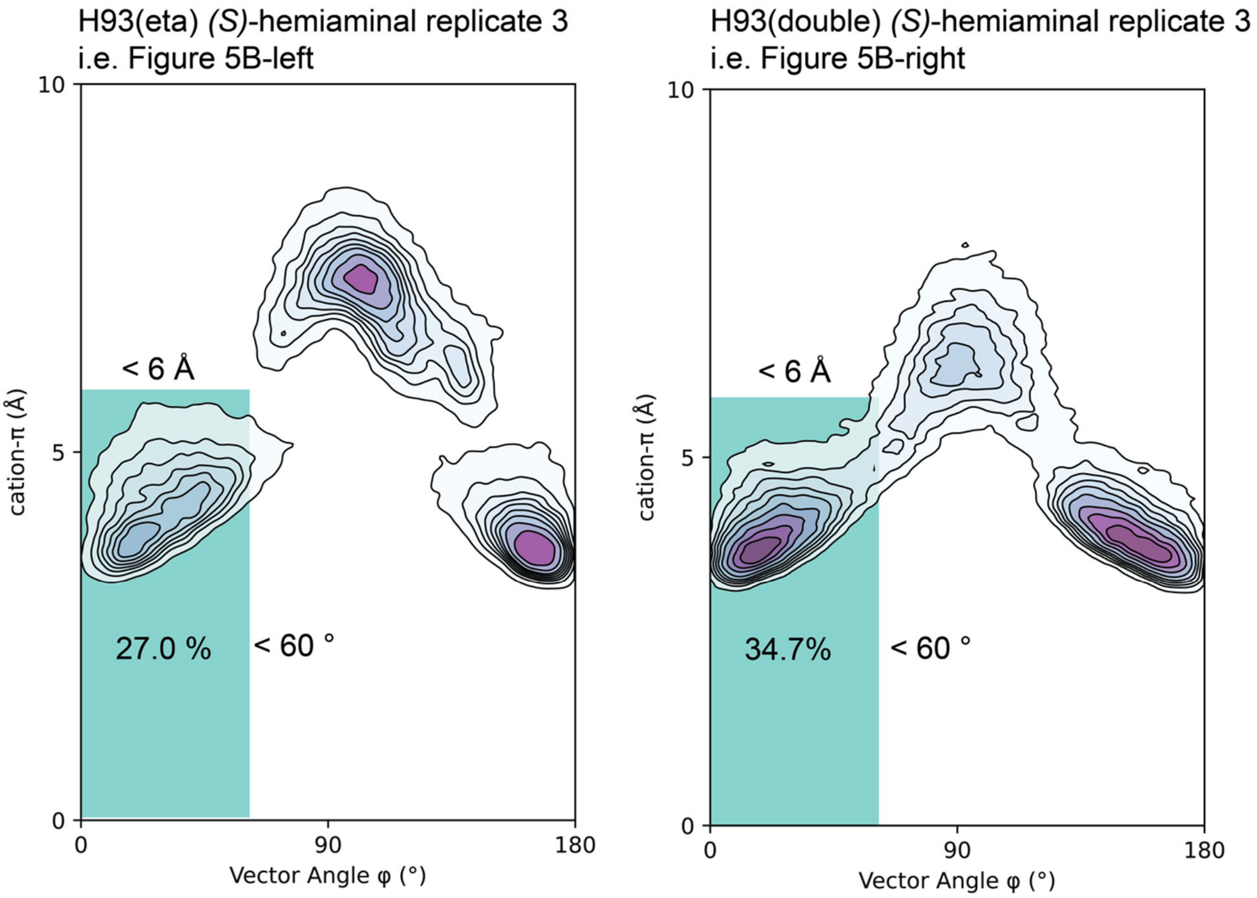

Figure S21: Measurement of cation-π interactions during the MD simulations depicted in main text Figure 5B. The angle of the dot-product between vectors projected perpendicular to the plane of the pAF ring and between the centre of the pAF ring and the central-carbon atom of the guanidium moiety of R92 is shown on the x-axis. The y-axis shows the distance between the centre of the pAF ring and the central-carbon atom of the guanidinium moiety of R92. Frames below the cutoff values (<60° and <6Å) are consistent with cation-π interactions, being 27.0% and 34.7% of the simulations, respectively.

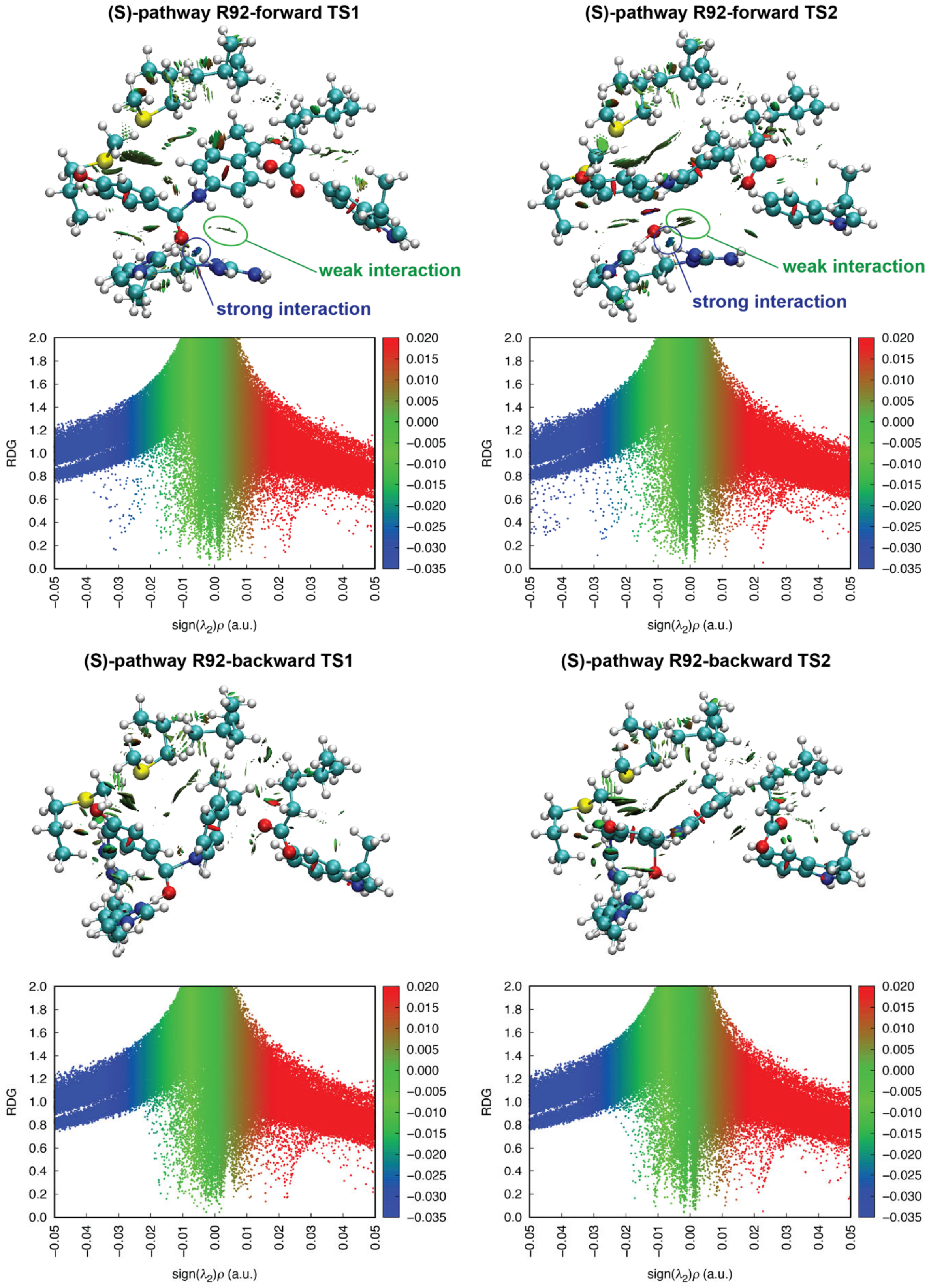

Figure S22: Non-covalent interaction (NCI) analysis for TS1 and TS2 of the quantum cluster for the R92-forward and R92-backward conformers of the *(S)*-configured pathway. The RDG plots demonstrate the presence of H-bonding interactions in the R92-forward conformation and not in the R92-backward pathway. These interactions are a single H-bond formed between R92 and the hemiaminal oxo-moiety, highlighted in blue. Furthermore, there is a limited region of weak-interaction between R92 and the pAF aromatic ring, which could be a cation-π interaction, highlighted in green.

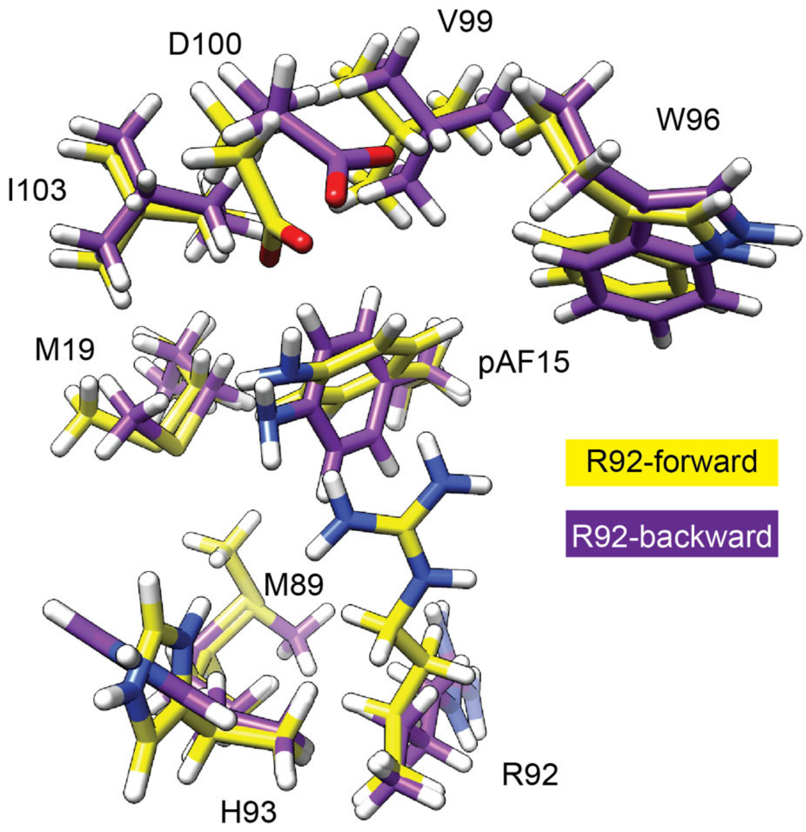

Figure S23: Overlay of the amino-acid residues employed in the two quantum cluster models taken from each monomer of the homodimer in the RMH crystal structure

Experimental Methods

1. *Materials and Equipment*

Chemicals were purchased from commercial suppliers (Sigma and TCI) and used without further purification. The unnatural amino acid pAzF was purchased as the enantiopure hydrochloride salt from Iris-Biotech. HPLC analysis was conducted using a Shimadzu LC-10ADVP HPLC equipped with a Shimadzu SPD-M10AVP diode array detector. Plasmid pEVOL-pAzF was obtained from Addgene (pEvol-pAzF was a gift from Prof. Peter Schultz (The Scripps Research Institute))[7]. Plasmid pEVOL_pAzFRS.2.t1 was obtained from Addgene (pEVOL_pAzFRS.2.t1 was a gift from Prof. Farren Isaacs, Yale University)[8]. *E. coli* strains NEB10-beta and BL21(DE3) (New England Biolabs) were used for cloning and expression, respectively. Primers were synthesized by Eurofins MWG Operon. PCR/Plasmid Purification Kits were obtained from QIAGEN and DNA sequencing carried out by Eurofins Genomics. Turbo Hot-Start polymerase and DpnI were purchased from New England Biolabs. Concentrations of DNA and protein solutions were determined based on the absorption at 260 nm or 280 nm on a Thermo Scientific Nanodrop 2000 UV-Vis spectrophotometer. UV/Vis absorption spectra and kinetic assays were recorded at 25 ºC on a Jasco V-660 spectrophotometer. Ampicillin and chloramphenical were used at final concentrations of 100 ug/mL and 34 ug/mL, respectively. Arabinose and IPTG were used to induce expression at final concentrations of 0.02% and 1 mM, respectively. cOmplete protease inhibitor cocktail tablets (Roche) were used during protein purification. DNAseI was purchased from Sigma as lyophilised powder.

2. *Quikchange Mutagenesis*

Most of the mutants used in this study were previously constructed[1,2,9,10]. The plasmids encoding LmrR_V15X_N19M_F93H, LmrR_V15X_L18R_M89N and LmrR_V15X_S95G_M89N were produced by Quikchange mutagenesis from LmrR_V15X_N19M, LmrR_V15X_L18R and LmrR_V15X_M89N templates, respectively following the manufacturer protocol using the Phusion HF polymerase (https://www.neb.com/en/protocols/0001/01/01/pcr-protocol-m0530; X=TAG amber stop codon). For the LmrR_V15X_N19M_F93H mutant, the reaction was performed in a 25 μL total volume with 20 annealing cycles at 55 °C. For the LmrR_V15X_L18R_M89N and LmrR_V15X_S95G_M89N mutants, the reaction was performed in a 50 μL total volume with 16 annealing cycles at 67 or 66 °C, respectively. The 1 uL of DpnI was added and the reactions were incubated at 37 °C for 1-1.5 hrs. In the case of the LmrR_V15X_L18R_M89N and LmrR_V15X_S95G_M89N mutants a PCR purification kit was then used according to the manufacturer protocol (Qiagen). The resulting solutions were used to transform chemically competent *E. coli* NEB10-beta) by heatshock at 42 °C for 30 seconds followed by recovery at 37 °C for 45 minutes in SOC medium (1 mL). The transformants thus obtained where harvested by centrifugation, resuspended in 100 μL SOC medium and plated onto LB agar plates containing ampicillin and incubated at 37 °C overnight. Three colonies were innocluted into 5 mL LB medium containing ampicillin and grown at 37 °C overnight with shaking at 135 rpm. The cells were harvested by centriguation and plasmid DNA was extracted and sequenced using T7 forward primers. Upon confirmation of successful mutagenesis, the same procedure was followed using the isolated plasmids as template and D59K_fwd and D59K_rev primers. Finally the plasmids containing both Q55K and D59K mutations according to sequencing results were transformed into chemically competent *E. coli* BL21(DE3) cells containing pEVOL_pAzF 42 °C for 30 seconds followed by recovery at 37 °C for 45 minutes in SOC medium (1 mL). The transformants thus obtained were harvested by centrifugation, resuspended in 100 μL SOC medium and plated onto LB agar plates containing ampicillin and chloramphenicol and incubated at 37 °C overnight. A single colony was innocluted into 5 mL LB medium containing ampicillin and chloramphenicol and grown at 37 °C overnight with shaking at 135 rpm. This was used to make a glycerol stock which was later used for protein production.

To revert the lysine knock-out mutations K55D and K59Q in order to obtain well-diffracting crystals of the evolved LmrR_pAF mutants, Quikchange mutagenesis was performed on pET17b+ constructs containing the LmrR plasmids either V15TAG_L18R_S95G_M89N or V15TAG_A92R_N19M_F93H mutations. First the Q55K mutation was reverted using Q55K_fwd and Q55K_rev primers. The reactions were conducted in 50 μL total volume with template plasmid DNA (50-150 ng), forward and reverse primers (2.5 μL each of a 10 μM stock solution), DMSO (1.5 μL), Pfu buffer (10x stock solution, 5 μL), turbo Hot-Start polymerase (1 μL) and nuclease-free water up to the total volume. The PCR reaction was then conducted in a thermocycler by initial denaturation at 95 °C for 3 minutes followed by 15 cycles of (1) denaturation at 95 °C for 30 seconds (2) annealing at (T_m-5) °C for 2 minutes (3) extension at 72 °C for 10 minutes then (4) a final extension step was conducted at 72 °C for 10 minutes. The reactions were then subject to DpnI digestion (1 μL) at 37 °C and then purified. The resulting product (7 μL) was then transformed into chemically competent *E. coli* NEB10-beta cells (50 μL aliqout) by heatshock at 42 °C for 30 seconds followed by recovery at 37 °C for 45 minutes in SOC medium (1 mL). The transformants thus obtained where harvested by centrifugation, resuspended in 100 μL SOC medium and plated onto LB agar plates containing ampicillin and incubated at 37 °C overnight. Three colonies were innocluted into 5 mL LB medium containing ampicillin and grown at 37 °C overnight with shaking at 135 rpm. The cells were harvested by centriguation and plasmid DNA was extracted and sequenced using T7 forward primers. Upon confirmation of successful mutagenesis, the same procedure was

followed using the isolated plasmids as template and D59K_fwd and D59K_rev primers. Finally the plasmids containing both Q55K and D59K mutations according to sequencing results were transformed into chemically competent *E. coli* BL21(DE3) cells containing pEVOL_pAzF 42 °C for 30 seconds followed by recovery at 37 °C for 45 minutes in SOC medium (1 mL). The transformants thus obtained were harvested by centrifugation, resuspended in 100 µL SOC medium and plated onto LB agar plates containing ampicillin and chloramphenicol and incubated at 37 °C overnight. A single colony was innocluted into 5 mL LB medium containing ampicillin and chloramphenicol and grown at 37 °C overnight with shaking at 135 rpm. This was used to make a glycerol stock which was later used for protein production.

Primer sequences (5' to 3'):

D55K_fwd	CCCTGTATACGATTTTTAAACGTCTGGA	T_m = 61.5 °C
D55K_rev	CGTCCTGTTCCAGACGTTTAAAAATC	T_m = 61.1 °C
Q59K_fwd	GTCTGGAAAAAGACGGCATTATCAGCTCT	T_m = 65.3 °C
Q59K_rev	TGCCGTCTTTTTCCAGACGTTTAAAAATCGTA	T_m = 65.8 °C
F93H_fwd	GCCTGGCGCATGAATCCTGGAGTCGT	T_m = 70.8 °C
F93H_rev	CAGGATTCATGCGCCAGGCGCATGTT	T_m = 70.3 °C
M89N_fwd#	GGCCATGAAAACAACCGCCTGGCGTTCGAATC	T_m = 73.1 °C
M89N_rev#	GATTCGAACGCCAGGCGGTTGTTTTCATGGCC	T_m = 73.1 °C
M89N_fwd2*	GGCCATGAAAACAACCGCCTGGCGTTCG	T_m = 72.4 °C
M89N_rev2*	CGAACGCCAGGCGGTTGTTTTCATGGCC	T_m = 72.4 °C

#Used for production of the LmrR_V15X_L18R_M89N mutant. *Used for production of the LmrR_V15X_S95G_M89N mutant.

3. Protein Production and Purification

Conducted as previously described[9]. Glycerol stocks of *E. coli* BL21(DE3) cells harbouring both pET17b+ vector containing the relevant LmrR mutant with amber stop codon (TAG) in position valine-15 as well as pEVOL_pAzF or pAzF_RS2.t1 were used to innoculate 5 mL LB medium containing ampicillin and chloramphenicol and at 37 °C overnight. The dense cultures were then diluted 200-fold into LB containing ampicillin and chloramphenicol (typically proteins were produced in cultures of 250 mL or 500 mL in Erlenmeyer flasks of 4 times the culture volume) and incubated at at 37 °C with shaking at 135 rpm. When the OD_{600} reached 0.8-1.2 expression was induced by addition of arabinose, IPTG and pAzF (final concentration 1 mM). Expression was conducted at 30 °C with shaking at 135 rpm for 16 hours .The cells were harvested by centrifugation at 6,000 rpm for 20 minutes at 4 °C and resuspended in buffer (50 mM NaH_2PO_4, 150 mM NaCl, pH = 8) containing protease inhibitor and the cells were lysed by sonication. DNaseI (one small spatula tip), and $MgCl_2$ (2 mM final concentration) were added and the disrupted cells were incubated on ice for 20 minutes. The cell debris was removed by centrifugation (12,000 rpm, 45 minutes at 4 °C) and the supernatant was applied to Strep-tactin columns and purified according to the manufacturer protocol. After purification, the protein was incubated overnight at 4 °C or for 1 hour at room temperature with TCEP (10 mM final concentration) to reduce the azido group. Finally, the excess TCEP and other buffer components were removed via dialysis or desalting with a PD-10 column (GE Healthcare). For proteins containing K55 and K59 residues, further purification was conducted using heparin column as previously described[11].

4. Crystallisation of proteins, data collection and refinement

LmrR variants RMH* and RGN* (the asterisk signifies that the variants carried the reverse mutations D55K and Q59K which support crystallization) were expressed and purified as described[12]. To improve purity and monodispersity, LmrR-RGN* was subjected to size exclusion chromatography as an extra purification step using a Superdex 200 column (Cytiva) and a running buffer containing 20 mM Tris-HCl, pH 8.0, 280 mM NaCl and 1 mM EDTA (TNE buffer). Purified proteins were concentrated to 7-8 mg/ml in TNE buffer and crystals were obtained by the sitting-drop vapour-diffusion method at 20 °C. Crystals of LmrR-RMH* grew against a reservoir solution containing 10-13% polyethylene glycol (PEG) 2000 monomethyl ether in 0.1 M Bis-Tris propane buffer, pH 8.5, while LmrR-RGN* crystals were obtained with 25% PEG 1500 in 0.1 M sodium propionate, sodium cacodylate trihydrate, Bis-Tris propane (PCTP) buffer, pH 7-8. Prior to flashcooling in liquid nitrogen, crystals were briefly soaked in a cryoprotectant solution of 25% glycerol, 15% PEG 2000 ME, 0-250 mM NaCl in 0.1 M PCTP, pH 7-8.5. X-ray diffraction data sets were collected on the MASSIF-1 and ID23-2 beamlines of the European Synchrotron Radiation Facility (ESRF), Grenoble. Diffraction data were processed with the program XDS[13] and scaled using AIMLESS[14] from the CCP4 software suite[15]. Molecular replacement was performed with PHASER[16], using PDB entry 6I8N as an initial search model. Automatic and manual rebuilding of the structures was carried out with BUCCANEER[17] and COOT[18], respectively, and initial model refinement with REFMAC5[19]. The final rounds of restrained refinement were performed with Phenix.refine[20]. Noncrystallographic symmetry and TLS were applied throughout the refinement. Water molecules were added following standard criteria based on electron density and proper hydrogen bond interaction geometries. Two final models were obtained for LmrR-RMH* with X-ray data sets extending to 2.24 Å and 2.55 Å resolution, collected from different crystals. The final model of LmrR-RGN* was refined to 2.45 Å resolution (see Table S3 for a summary of the crystallographic statistics). The final models were validated using MolProbity[21]. Coordinates and structure factors are available at the PDB under accession codes 9GKR (variant RMH*, crystal 1), 9GKS (variant RMH*, crystal 2) and 9GKT (variant RGN*).

5. *Ensemble Refinement*

Ensemble refinement was performed as described[22] using the latest version of PHENIX[23]. The structure of LmrR_V15pAF (PDB entry 6I8N[1], the parent of the variants LmrR-RMH and LmrR-RGN) was first rerefined with Phenix.refine against the original X-ray data set. For all structures, the contribution of lattice disorder was modelled by a TLS model containing one TLS group per chain. Parameters pTLS, wxray_coupled_tbath_offset and tx were optimized by running multiple ensemble refinement jobs, as suggested in the documentation.

Computational Methods

1. *DFT calculations*

Calculations were conducted at DFT level using Gaussian 16[24] software. Structures were optimised using the B3LYP 6-31G (d,p)[25,26] basis set with either water (epsilon=78.35, for the truncated Friedel-Crafts reaction model) or chlorobenzene (epsilon=5.70, for the QM clutser calculations) implicit solvent models (SMD model[27]). Transition states were identified by the presence of an imaginary frequency vibration along the reaction coordinate, whilst stationary points were confirmed to contain no imaginary frequencies. For the truncated model of the Friedel-Crafts reaction, intrinsic reaction coordinate calculations were performed to confirm the connection of the transition state with the expected reactants and products. For the quantum cluster calculations, the energies were then refined with an extended basis set, B3LYP 6-311G++(2d,2p)[28,29]. NCI analysis was conducted with the MultiWFN software package[30,31] according to the published method[32] and visualised with VMD[33] and gnuplot.

2. *Molecular Docking*

Docking simulations were conducted using GOLD[34]. The missing loops in the protein structure were modelled using the MODELLER package integrated into USCF Chimera[35]. Missing terminal residues in the crystal structure were omitted from the simluations. Structures of the pAF residue in substrate-bound states (either with hexenal-iminium ion, or para-hydroxy benzaldehyde hemiaminal derivatisations) containing N-acetyl and C-amido modifications (to mimic the protein backbone) were optimised using Gaussian. The pAF side chain was then removed from the scaffold structure, and the main-chain of the substrate-bound residue was removed (except C_α) then covalent docking was conducted between the C_α atom of the scaffold and ligand. The genetic algorithm parameters were set to 50 runs and a minimum of 100,000 operations and early termination was not allowed to ensure 50 solutions were produced. The evaluation sphere was set to 12 Å and Key residues around the binding site were allowed to be flexible according to the inbuilt rotamer library and the rotamers present in the crystal structure were further added to remove potential bias. The solutions were scored and ranked with GOLDscore. Non-covalent dockings were run with 2-methyl indole on the top ranked solution with the hexenal iminium ion following a similar procedure to obtain the starting points for the Friedel-Crafts NAC simulations.The top solution as ranked by GOLDscore[36] was used as the starting point for MD simulations, except for the simulations of LmrR_pAF_RMH with the ε-protonation state of H93, where H-bonding interactions between W96 and the hemiaminal hydroxy moiety persisted throughout most replicates and were not percieved as informative for catalysis.In this case, the next highest ranked solution by GOLDscore was used where these interactions were not present.

3. *Molecular Dynamics Simulations*

All simulations were prepared using xleap by emmbeding the systems in a cubic water box under periodic boundary conditions and neutralizing them with Na^+ and Cl^- ions. The protein system was parametrized with the AMBER14SB[37] forcefield, and TIP3P[38] was used for water. The non-proteogenic components (non-canonical residues and other ligands) were subjected to RESP (restrained electrostatic potential) calculations to obtain the charges and parameterised with GAFF[39] forcefield using amber tools. The resulting structures were then positioned together with the protein scaffold to replicate the docking pose as closely as geometrically feasible whilst maintaining the backbone configuration determined with DFT energy minimisation.

Thereafter molecular dynamics were run using the AMBER 22[40] software package. The simulation protocol consisted of sequential energy minimizations, first restricting the protein, then the backbone, and finally the ligands to avoid steric clashes and gradually relax the systems. This was

followed by a heating step, then NVT and NPT equilibration steps with backbone restraints. Finally, production simulations were run for 500ns, with six replicates to ensure a thorough sampling. The simulations were conducted under constant pressure using a barostat of 1 atm and constant tempertaure of 300K, controlled by a Langevin thermostat. The Langevin integrator was used with a time step of 2 fs and the SHAKE[41] algorithm was employed to constrain bonds that involve hydrogen. A cut-off of 8 Å was used for non-bonded interactions (short-range electrostatic and van der Waals interactions) and the PME[42] method was applied for long-range electrostatic interactions.

All MD trajectories were processed using cpptraj[4] and the convergance of the simulations was assesed by stabilisation of a set of analysis: RMSD, all-to-all RMSD, cluster-counting method[43] and PCA analysis.

4. ***Analysis of MD Simluations***

All analyses were carried out using cpptraj[4]. Hydrogen bonding interactions were counted setting the protein residues as potential donors/acceptors and the hemiaminal oxygen/hydroxyl-H as the acceptor/donor using cpptraj hbond tool. Dihedral angles and distances for the Friedel-Crafts NAC simulations were also analysed using cpptraj, as were the distance measurements for the hemiaminal MD-simluations. The density plots were produced using the seaborn[44] python package using the kdeplot functionality. Cluster analysis was conducted using cpptraj with the keywords “hieragglo clusters 10 averagelinkage” to produce a total of ten clusters. The atoms selected for the analysis were either the C-alpha atoms of the system, or else catalytically relevant atoms (specified in figure captions). All frames were used for the analysis.

References

(1) Mayer, C.; Dulson, C.; Reddem, E.; Thunnissen, A.-M. W. H.; Roelfes, G. Directed Evolution of a Designer Enzyme Featuring an Unnatural Catalytic Amino Acid. *Angew. Chem. Int. Ed.* **2019**, *58*, 2083–2087.

(2) Leveson-Gower, R. B.; Zhou, Z.; Drienovská, I.; Roelfes, G. Unlocking Iminium Catalysis in Artificial Enzymes to Create a Friedel–Crafts Alkylase. *ACS Catal.* **2021**, *11* (12), 6763–6770. https://doi.org/10.1021/acscatal.1c00996.

(3) Casilli, F.; Canyelles-Niño, M.; Roelfes, G.; Alonso-Cotchico, L. Computation-Guided Engineering of Distal Mutations in an Artificial Enzyme. *Faraday Discuss.* **2024**, 10.1039.D4FD00069B. https://doi.org/10.1039/D4FD00069B.

(4) Roe, D. R.; Cheatham, T. E. PTRAJ and CPPTRAJ: Software for Processing and Analysis of Molecular Dynamics Trajectory Data. *J. Chem. Theory Comput.* **2013**, *9* (7), 3084–3095. https://doi.org/10.1021/ct400341p.

(5) Smith, R. H. B.; Dar, A. C.; Schlessinger, A. PyVOL: A PyMOL Plugin for Visualization, Comparison, and Volume Calculation of Drug-Binding Sites. bioRxiv October 24, 2019, p 816702. https://doi.org/10.1101/816702.

(6) McGibbon, R. T.; Beauchamp, K. A.; Harrigan, M. P.; Klein, C.; Swails, J. M.; Hernández, C. X.; Schwantes, C. R.; Wang, L.-P.; Lane, T. J.; Pande, V. S. MDTraj: A Modern Open Library for the Analysis of Molecular Dynamics Trajectories. *Biophysical Journal* **2015**, *109* (8), 1528–1532. https://doi.org/10.1016/j.bpj.2015.08.015.

(7) Chin, J. W.; Santoro, S. W.; Martin, A. B.; King, D. S.; Wang, L.; Schultz, P. G. Addition of P-Azido-l-Phenylalanine to the Genetic Code of Escherichia Coli. *J. Am. Chem. Soc.* **2002**, *124* (31), 9026–9027. https://doi.org/10.1021/ja027007w.

(8) Amiram, M.; Haimovich, A. D.; Fan, C.; Wang, Y.-S.; Aerni, H.-R.; Ntai, I.; Moonan, D. W.; Ma, N. J.; Rovner, A. J.; Hong, S. H.; Kelleher, N. L.; Goodman, A. L.; Jewett, M. C.; Söll, D.; Rinehart, J.; Isaacs, F. J. Evolution of Translation Machinery in Recoded Bacteria Enables Multi-Site Incorporation of Nonstandard Amino Acids. *Nat Biotechnol* **2015**, *33* (12), 1272–1279. https://doi.org/10.1038/nbt.3372.

(9) Drienovská, I.; Mayer, C.; Dulson, C.; Roelfes, G. A Designer Enzyme for Hydrazone and Oxime Formation Featuring an Unnatural Catalytic Aniline Residue. *Nat. Chem.* **2018**, *10*, 946–952.

(10) Leveson-Gower, R. B.; Boer, R. M.; Roelfes, G. Tandem Friedel-Crafts-Alkylation-Enantioselective-Protonation by Artificial Enzyme Iminium Catalysis. *ChemCatChem* **2022**, *14* (8). https://doi.org/10.1002/cctc.202101875.

(11) Gutiérrez de Souza, C.; Bersellini, M.; Roelfes, G. Artificial Metalloenzymes Based on TetR Proteins and Cu(II) for Enantioselective Friedel-Crafts Alkylation Reactions. *ChemCatChem* **2020**, *12* (12), 3190–3194. https://doi.org/10.1002/cctc.202000245.

(12) Villarino, L.; Splan, K. E.; Reddem, E.; Alonso-Cotchico, L.; Lledós, A.; Gutiérrez de Souza, C.; Thunnissen, A.-M. W. H.; Maréchal, J.-D.; Roelfes, G. An Artificial Heme Enzyme for Cyclopropanation Reactions. *Angew. Chem. Int. Ed.* **2018**, *57*, 7785–7789.

(13) Kabsch, W. Integration, Scaling, Space-Group Assignment and Post-Refinement. *Acta Crystallogr D Biol Crystallogr* **2010**, *66* (2), 133–144. https://doi.org/10.1107/S0907444909047374.

(14) Evans, P. R.; Murshudov, G. N. How Good Are My Data and What Is the Resolution? *Acta Crystallogr D Biol Crystallogr* **2013**, *69* (7), 1204–1214. https://doi.org/10.1107/S0907444913000061.

(15) Winn, M. D.; Ballard, C. C.; Cowtan, K. D.; Dodson, E. J.; Emsley, P.; Evans, P. R.; Keegan, R. M.; Krissinel, E. B.; Leslie, A. G. W.; McCoy, A.; McNicholas, S. J.; Murshudov, G. N.; Pannu, N. S.; Potterton, E. A.; Powell, H. R.; Read, R. J.; Vagin, A.; Wilson, K. S. Overview of the *CCP* 4 Suite and Current Developments. *Acta Crystallogr D Biol Crystallogr* **2011**, *67* (4), 235–242. https://doi.org/10.1107/S0907444910045749.

(16) McCoy, A. J.; Grosse-Kunstleve, R. W.; Adams, P. D.; Winn, M. D.; Storoni, L. C.; Read, R. J. *Phaser* Crystallographic Software. *J Appl Crystallogr* **2007**, *40* (4), 658–674. https://doi.org/10.1107/S0021889807021206.

(17) Cowtan, K. The *Buccaneer* Software for Automated Model Building. 1. Tracing Protein Chains. *Acta Crystallogr D Biol Crystallogr* **2006**, *62* (9), 1002–1011. https://doi.org/10.1107/S0907444906022116.

(18) Emsley, P.; Cowtan, K. *Coot* : Model-Building Tools for Molecular Graphics. *Acta Crystallogr D Biol Crystallogr* **2004**, *60* (12), 2126–2132. https://doi.org/10.1107/S0907444904019158.

(19) Murshudov, G. N.; Skubák, P.; Lebedev, A. A.; Pannu, N. S.; Steiner, R. A.; Nicholls, R. A.; Winn, M. D.; Long, F.; Vagin, A. A. *REFMAC* 5 for the Refinement of Macromolecular Crystal Structures. *Acta Crystallogr D Biol Crystallogr* **2011**, *67* (4), 355–367. https://doi.org/10.1107/S0907444911001314.

(20) Afonine, P. V.; Grosse-Kunstleve, R. W.; Echols, N.; Headd, J. J.; Moriarty, N. W.; Mustyakimov, M.; Terwilliger, T. C.; Urzhumtsev, A.; Zwart, P. H.; Adams, P. D. Towards Automated Crystallographic Structure Refinement with *Phenix.Refine*. *Acta Crystallogr D Biol Crystallogr* **2012**, *68* (4), 352–367. https://doi.org/10.1107/S0907444912001308.

(21) Williams, C. J.; Headd, J. J.; Moriarty, N. W.; Prisant, M. G.; Videau, L. L.; Deis, L. N.; Verma, V.; Keedy, D. A.; Hintze, B. J.; Chen, V. B.; Jain, S.; Lewis, S. M.; Arendall, W. B.; Snoeyink, J.; Adams, P. D.; Lovell, S. C.; Richardson, J. S.; Richardson, D. C. MolProbity: More and Better Reference Data for Improved All-Atom Structure Validation: PROTEIN SCIENCE.ORG. *Protein Science* **2018**, *27* (1), 293–315. https://doi.org/10.1002/pro.3330.

(22) Burnley, B. T.; Afonine, P. V.; Adams, P. D.; Gros, P. Modelling Dynamics in Protein Crystal Structures by Ensemble Refinement. *eLife* **2012**, *1*, e00311. https://doi.org/10.7554/eLife.00311.

(23) Liebschner, D.; Afonine, P. V.; Baker, M. L.; Bunkóczi, G.; Chen, V. B.; Croll, T. I.; Hintze, B.; Hung, L.-W.; Jain, S.; McCoy, A. J.; Moriarty, N. W.; Oeffner, R. D.; Poon, B. K.; Prisant, M. G.; Read, R. J.; Richardson, J. S.; Richardson, D. C.; Sammito, M. D.; Sobolev, O. V.; Stockwell, D. H.; Terwilliger, T. C.; Urzhumtsev, A. G.; Videau, L. L.; Williams, C. J.; Adams, P. D. Macromolecular Structure Determination Using X-Rays, Neutrons and Electrons: Recent Developments in *Phenix*. *Acta Crystallogr D Struct Biol* **2019**, *75* (10), 861–877. https://doi.org/10.1107/S2059798319011471.

(24) Gaussian 16.

(25) Ditchfield, R.; Hehre, W. J.; Pople, J. A. Self-Consistent Molecular-Orbital Methods. IX. An Extended Gaussian-Type Basis for Molecular-Orbital Studies of Organic Molecules. *The Journal of Chemical Physics* **1971**, *54* (2), 724–728. https://doi.org/10.1063/1.1674902.

(26) Hehre, W. J.; Ditchfield, R.; Pople, J. A. Self—Consistent Molecular Orbital Methods. XII. Further Extensions of Gaussian—Type Basis Sets for Use in Molecular Orbital Studies of Organic Molecules. *The Journal of Chemical Physics* **1972**, *56* (5), 2257–2261. https://doi.org/10.1063/1.1677527.

(27) Marenich, A. V.; Cramer, C. J.; Truhlar, D. G. Universal Solvation Model Based on Solute Electron Density and on a Continuum Model of the Solvent Defined by the Bulk Dielectric Constant and Atomic Surface Tensions. *J. Phys. Chem. B* **2009**, *113* (18), 6378–6396. https://doi.org/10.1021/jp810292n.

(28) McLean, A. D.; Chandler, G. S. Contracted Gaussian Basis Sets for Molecular Calculations. I. Second Row Atoms, Z=11–18. *The Journal of Chemical Physics* **1980**, *72* (10), 5639–5648. https://doi.org/10.1063/1.438980.

(29) Krishnan, R.; Binkley, J. S.; Seeger, R.; Pople, J. A. Self-consistent Molecular Orbital Methods. XX. A Basis Set for Correlated Wave Functions. *The Journal of Chemical Physics* **1980**, *72* (1), 650–654. https://doi.org/10.1063/1.438955.

(30) Lu, T.; Chen, F. Multiwfn: A Multifunctional Wavefunction Analyzer. *J Comput Chem* **2012**, *33* (5), 580–592. https://doi.org/10.1002/jcc.22885.

(31) Lu, T. A Comprehensive Electron Wavefunction Analysis Toolbox for Chemists, Multiwfn. *The Journal of Chemical Physics* **2024**, *161* (8), 082503. https://doi.org/10.1063/5.0216272.

(32) Johnson, E. R.; Keinan, S.; Mori-Sánchez, P.; Contreras-García, J.; Cohen, A. J.; Yang, W. Revealing Noncovalent Interactions. *J. Am. Chem. Soc.* **2010**, *132* (18), 6498–6506. https://doi.org/10.1021/ja100936w.

(33) Humphrey, W.; Dalke, A.; Schulten, K. VMD: Visual Molecular Dynamics. *Journal of Molecular Graphics* **1996**, *14* (1), 33–38. https://doi.org/10.1016/0263-7855(96)00018-5.

(34) Jones, G.; Willett, P.; Glen, R. C.; Leach, A. R.; Taylor, R. Development and Validation of a Genetic Algorithm for Flexible Docking. *Journal of Molecular Biology* **1997**, *267* (3), 727–748. https://doi.org/10.1006/jmbi.1996.0897.

(35) Yang, Z.; Lasker, K.; Schneidman-Duhovny, D.; Webb, B.; Huang, C. C.; Pettersen, E. F.; Goddard, T. D.; Meng, E. C.; Sali, A.; Ferrin, T. E. UCSF Chimera, MODELLER, and IMP: An Integrated Modeling System. *Journal of Structural Biology* **2012**, *179* (3), 269–278. https://doi.org/10.1016/j.jsb.2011.09.006.

(36) Verdonk, M. L.; Cole, J. C.; Hartshorn, M. J.; Murray, C. W.; Taylor, R. D. Improved Protein–Ligand Docking Using GOLD. *Proteins: Structure, Function, and Bioinformatics* **2003**, *52* (4), 609–623. https://doi.org/10.1002/prot.10465.

(37) Maier, J. A.; Martinez, C.; Kasavajhala, K.; Wickstrom, L.; Hauser, K. E.; Simmerling, C. ff14SB: Improving the Accuracy of Protein Side Chain and Backbone Parameters from ff99SB. *J. Chem. Theory Comput.* **2015**, *11* (8), 3696–3713. https://doi.org/10.1021/acs.jctc.5b00255.

(38) Jorgensen, W. L.; Chandrasekhar, J.; Madura, J. D.; Impey, R. W.; Klein, M. L. Comparison of Simple Potential Functions for Simulating Liquid Water. *The Journal of Chemical Physics* **1983**, *79* (2), 926–935. https://doi.org/10.1063/1.445869.

(39) Wang, J.; Wang, W.; Kollman, P. A.; Case, D. A. Automatic Atom Type and Bond Type Perception in Molecular Mechanical Calculations. *Journal of Molecular Graphics and Modelling* **2006**, *25* (2), 247–260. https://doi.org/10.1016/j.jmgm.2005.12.005.

(40) Case, D. A.; Aktulga, H. M.; Belfon, K.; Ben-Shalom, I. Y.; Berryman, J. T.; Brozell, S. R.; Cerutti, D. S.; Cheatham, T. E. I.; Cisneros, G. A.; Cruzeiro, V. W. D.; Darden, T. A.; Forouzesh, N.; Ghazimirsaeed, M.; Giambaşu, G.; Giese, T. J.; Gilson, M. K.; Gohlke, H.; Goetz, A. W.; Harris, J.; Huang, Z.; Izadi, S.; Izmailov, S. A.; Kasavajhala, K.; Kaymak, M. C.; Kovalenko, A.; Kurtzman, T.; Lee, T. S.; Li, P.; Li, Z.; Lin, C.; Liu, J.; Luchko, T.; Luo, R.; Machado, M. R.; Manathunga, M.; Merz, K. M. Jr.; Miao, Y.; Mikhailovskii, O.; Monard, G.; Nguyen, H. M.; O'Hearn, K. A.; Onufriev, A. V.; Pan, F.; Pantano, S.; Rahnamoun, A.; Roe, D. R.; Roitberg, A.; Sagui, C.; Schott-Verdugo, S.; Shajan, A.; Shen, J.; Simmerling, C.; Skrynnikov, N. R.; Smith, J.; Swails, J.; Walker, R. C.; Wang, J.; Wu, X.; Wu, Y.; Xiong, Y.; Xue, Y.; York, D. M.; Zhao, C.; Zhu, Q.; Kollman, P. A. Amber 2024, 2024.

(41) Ryckaert, J.-P.; Ciccotti, G.; Berendsen, H. J. C. Numerical Integration of the Cartesian Equations of Motion of a System with Constraints: Molecular Dynamics of *n*-Alkanes. *Journal of Computational Physics* **1977**, *23* (3), 327–341. https://doi.org/10.1016/0021-9991(77)90098-5.

(42) Essmann, U.; Perera, L.; Berkowitz, M. L.; Darden, T.; Lee, H.; Pedersen, L. G. A Smooth Particle Mesh Ewald Method. *The Journal of Chemical Physics* **1995**, *103* (19), 8577–8593. https://doi.org/10.1063/1.470117.

(43) Smith, L. J.; Daura, X.; van Gunsteren, W. F. Assessing Equilibration and Convergence in Biomolecular Simulations. *Proteins* **2002**, *48* (3), 487–496. https://doi.org/10.1002/prot.10144.

(44) Waskom, M. L. Seaborn: Statistical Data Visualization. *Journal of Open Source Software* **2021**, *6* (60), 3021. https://doi.org/10.21105/joss.03021.
